# Lay representations of social class: A mixed methods approach to wealth‐based group perceptions and stereotypes

**DOI:** 10.1111/bjso.70003

**Published:** 2025-06-27

**Authors:** Ángel del Fresno‐Díaz, Efraín García‐Sánchez, Elena Padial‐Rojas, Guillermo B. Willis, Soledad de Lemus

**Affiliations:** ^1^ Department of Social Psychology University of Granada Granada Spain; ^2^ Institute of Psychology University of Gdańsk Gdańsk Poland; ^3^ Stanford SPARQ Stanford University Stanford California USA

**Keywords:** mix method, social class, social stratification, wealth‐based groups, wealth‐based stereotyping

## Abstract

People's perceptions of social classes may differ from scholars' definitions. We used a mixed method, sensitive to context, to examine lay perceptions of social classes in Spain. In Study 1 (*N* = 90), we conducted qualitative interviews to examine how people spontaneously characterize wealth‐based groups. Participants identified between two and seven groups. We grouped these into five main analytical categories for analytical purposes: poor, lower and working classes, middle classes, upper classes and rich and beyond. These groups were described based on material characteristics, traits and culture. Positive traits were mainly associated with non‐wealthy groups, especially the lower and working classes, while negative traits were associated with wealthy groups. In Studies 2 (*N* = 251) and 3 (*N* = 190), we extended these findings quantitatively, showing that positive stereotypes were associated with non‐wealthy groups, whereas negative stereotypes were associated with wealthy groups. Using psychometric networks, non‐wealthy groups were ascribed more positive traits—with some ambivalences—while wealthy groups were mainly described using negative traits. We confirmed this pattern of results through meta‐analyses. These findings highlight the importance of lay perspectives in theoretical frameworks and the need for context‐sensitive approaches in analysing social class representations.

## INTRODUCTION

How do individuals psychologically perceived, evaluate and categorize social groups based on perceived wealth differences? Research on social class stereotypes (e.g. Durante et al., 2017; Fiske et al., 2002) has often relied on theoretical definitions of social class developed by scholars rather than on people's lay beliefs about how economic differences shape social categories. Lay theories refer to organized structures of knowledge that are not necessarily anchored in formal theories about social groups (Hong et al., 2003). This distinction is important because, while social class is often conceptualized as a multidimensional construct that includes economic, cultural and social factors (Bourdieu, 1979/1984; Kraus et al., 2012), individuals may spontaneously categorize others primarily based on wealth. Wealth thus serves as a salient cue for group perception (Jetten et al., 2017), shaping how people intuitively structure social categories.

Moreover, lay people's views of different wealth‐based groups have significant implications for how individuals behave towards them (Lickel et al., 2003). Research on stereotype content has shown that attitudes towards wealth‐based groups tend to be negative towards the poor, mostly positive towards the middle class and ambivalent towards the rich (Cuddy et al., 2008; Fiske et al., 2002; Fiske & Markus, 2012). However, some studies offer a different perspective, suggesting that the poor, as well as the working class, are evaluated more positively than the rich (Durante et al., 2017; Kervyn et al., 2010). Importantly, the tripartite conception of class—poor, middle class and rich—and attributes associated with these groups may not be universal and can vary across different cultural contexts beyond the United States.

This paper approaches the study of social class representations by examining how individuals naturally perceive and categorize social groups based on wealth (Study 1) and how stereotypes are attributed to these wealth‐based groups (Studies 2 and 3) in Spain. Social class is thus the theoretical concept, while wealth‐based groups provide the empirical and descriptive approach to that concept. Specifically, we adopt a psychological perspective that emphasizes subjective perception (García‐Sánchez et al., 2018; Willis et al., 2022). This perspective focuses on how individuals spontaneously categorize groups based on wealth and the stereotypes associated with these categories (Jetten et al., 2021). While these wealth‐based groupings may sometimes overlap with conventional social class categories, they primarily reflect how people perceive economic stratification in their context. To fill the knowledge gap on how lay theories define social class, this paper qualitatively explores wealth‐based categorizations, identifying context‐specific views of social class, categorizations and stereotypes as perceived by individuals, rather than conforming to pre‐existing models. Additionally, we quantitatively examine the structure and valence of stereotypes attributed to wealth‐based groups.

### Lay perceptions of wealth‐based groups capture social class

People's perceptions of wealth‐based groups are shaped by a range of factors, including how they understand socio‐economic or cultural differences between groups. Although social class and socio‐economic status (SES) are distinct constructs, they are closely related (Antonoplis, 2023; Easterbrook et al., 2023). Social class includes identity, socialization and cultural dimensions (Becker et al., 2017; Manstead, 2018), whereas SES refers to individuals' access to valuable resources, such as income, education and occupational prestige (Kraus et al., 2017). However, people's lay beliefs often conflate social class and SES when thinking about wealth‐based groups. For example, when considering poverty, people draw on lay beliefs that frame poverty as a broader experience of deprivation, including limited access to food, housing and social opportunities (García‐Castro et al., 2022), rather than thinking in terms of absolute income, such as living on less than $2 a day (e.g. World Economic Forum., 2019). Thus, lay beliefs about wealth‐based groups reflect both the implications of SES and the perceived characteristics associated with social class. These perceptions show that wealth operates as a central element in social categorization. In this sense, wealth‐based groups provide an empirical and descriptive approach to representations of social class, as wealth itself may be a sufficient cue for categorization (Jetten et al., 2017).

Perceived wealth serves as a key cue people use to classify others into different social groups (Jetten et al., 2017, 2021). The labels and characteristics attributed to groups differing in perceived wealth shape how individuals understand social class, social stratification and economic inequality (Peters & Jetten, 2023; Willis et al., 2022). From a social identity approach, the salience, accessibility and relevance of wealth contribute to the formation of social categories through social comparison processes, establishing norms that reinforce group distinctions and stereotypes (Jetten et al., 2017; Turner et al., 1987). This suggests that wealth—independent of other SES components—is a psychologically meaningful basis for categorizing others into social groups and understanding social stratification.

Although wealth exists on a continuum, both academics and laypeople tend to segment it into distinct groups, using commonly shared labels in everyday discourse (Horwitz & Dovidio, 2017). In the United States, for example, terms such as ‘rich’, ‘middle class’ and ‘poor’ are widely recognized and commonly used (Pew Research Center, 2012). Others such as ‘lower class’, ‘working class’ or ‘upper class’ are employed in different cultural contexts (e.g. the United Kingdom; Elenbaas et al., 2024; Evans & Mellon, 2016), reflecting alternative ways of dividing society along wealth lines. While some classifications rely on economic thresholds, others are shaped by broader perceptions of status and lifestyle, which merge in both social narratives and individual perceptions. For example, individuals often rely on visible cues of wealth to categorize others (Rinn et al., 2023). Given that these categorizations may emerge from intuitive judgements rather than strict economic standards, we argue that examining the labels and criteria that individuals spontaneously use to structure social groups based on wealth in a context‐sensitive way can provide deeper insights into subjective representations of social classes.

### Wealth‐based evaluations: Attitudes and stereotypes

The content of wealth stereotypes provides a framework for understanding how wealth‐based groups are represented in people's minds (Jetten et al., 2017). One of the theoretical frameworks used to study social group stereotypes is the stereotype content model (SCM), which conceptualizes stereotypes along the dimensions of warmth and competence (Fiske et al., 2002). Warmth reflects interpersonal sympathy, while competence denotes the ability to achieve goals, both of which stem from perceived SES and interdependence (cooperative‐competitive; Fiske et al., 2002, 2007). Stereotypes may be either unambiguously positive or negative: high‐status and cooperative groups are perceived as both competent and warm, whereas low‐status and competitive groups are seen as incompetent and cold (with some cultural variations; Cuddy et al., 2009). However, stereotypes can also be ambivalent, with the rich often viewed as competent but cold, and the poor as warmer yet less competent (Durante et al., 2017; Fiske et al., 2002).

These ambivalent attitudes towards wealth‐based groups are influenced by contextual factors, such as income inequality (Durante et al., 2013). Experimentally, high‐income individuals are considered more competent when portrayed in high inequality contexts (Connor et al., 2021), and both rich and poor are seen as less competent overall (Tanjitpiyanond et al., 2022). These dynamics align with the vertical dimension of the social evaluation model, which focuses on group competence and assertiveness (Abele et al., 2021). In contrast, the horizontal dimension, which concerns group friendliness and morality, often portrays wealthy individuals as lacking moral traits (Moreno‐Bella et al., 2019). Poor individuals are generally perceived as friendlier and more moral than the rich (Kervyn et al., 2010), yet both groups may be viewed as immoral in unequal contexts (Tanjitpiyanond et al., 2022).

Wealth‐based stereotypes have been extensively studied, particularly in capitalist societies such as the United States, where wealth is seen as merit‐based, and poverty is stigmatized—as opposed to post‐communist societies (Grigoryan et al., 2020). This raises the question of whether individuals in different contexts, such as Spain, conceptualize wealth‐based groups in the same way as existing models suggest (Durante et al., 2013; SCM, Fiske et al., 2002, 2007). Furthermore, while previous research has primarily focused on evaluating individuals at the extremes of wealth, such as rich or poor individuals (Durante et al., 2013, 2017; Tanjitpiyanond et al., 2023), less attention has been paid to how people represent a broader range of intermediate wealth‐based group. These include categories identified qualitatively by participants, such as homeless, poor, lower class, working class, lower‐middle class, middle class, upper‐middle class, upper class, rich, elite, 1%, nobility or royalty (Elenbaas et al., 2024). Given that the literature emphasizes subjective measurements (e.g. Davidai & Wienk, 2021; García‐Sánchez et al., 2018), people's views on social stratification may not align with class narratives constructed by formal theoretical approaches.

## THE PRESENT RESEARCH

This paper addresses two main research questions: (1) How individuals mentally structure wealth‐based groups beyond pre‐established categories and (2) How individuals perceive wealth‐based groups. Since individuals do not necessarily rely on formal definitions when categorizing others, examining the categories they spontaneously use can provide insights into how they subjectively structure social classes. Therefore, our first main objective is to study people's lay perceptions of wealth‐based groups by qualitatively exploring the labels and traits that individuals use to distinguish wealth‐based groups. In Study 1, we address this goal by adopting a bottom‐up qualitative approach, in which we collect wealth‐based group labels, their defining characteristics and stereotype‐related traits through open‐ended questions.

The second objective is to investigate the structure and content of wealth‐based stereotypes. To achieve this goal, we first analyse the spontaneous characterization of self‐generated wealth groups in Study 1, and then further examine their content, valence and the links between different traits in Studies 2 and 3. In Study 2, we use participant‐generated labels and traits to quantitatively examine the attitudes and structure of wealth‐based stereotypes through psychometric networks. Such analysis provides higher ecological validity, however, self‐generated group labels can be biased in terms of valence, influencing participants' responses. Finally, in Study 3, we overcome this methodological limitation of Study 2 by presenting more neutral stimuli to frame the wealth‐based groups and ensuring the robustness of our findings with meta‐analyses of the association between wealth‐based groups and stereotype valence.

## STUDY 1

In Study 1 (https://osf.io/9wzmj/?view_only=8c5e62f11f774053b80b35e124ec101f), we conducted qualitative research using semi‐structured interviews to explore how participants naturally categorize groups based on wealth. In addition, we explored associations between wealth‐based groups and their associated traits.

### Method

#### Participants

Data were collected from 90 participants, including undergraduate students from the University of Granada (studying psychology and occupational therapy) and their acquaintances from the general population. The sample consisted of 72 women and 18 men, aged between 18 and 31 years (M = 19.48; SD = 2.18), with an average political ideology of centre‐left (M = 35.22; SD = 24.63, on a scale from 0 = ‘left/progressist’ to 100 = ‘right/conservative’) and an average subjective SES of M = 5.73 (SD = 1.06) on a scale of 1–10. Monthly household income ranged from 651 to 1131€ (11.1%), 1131 to 1951€ (31.5%), 1951 to 2600€ (22.2%), and 2601 to 3016€ (10.1%). Most participants (74.4%) were undergraduates, with others having completed secondary education (1.1%), vocational training (10.1%), high school studies (5.6%), university studies (2.2%), master's degree (4.4%) or a Ph.D. (1.1%).

#### Procedure

**FIGURE 1:**
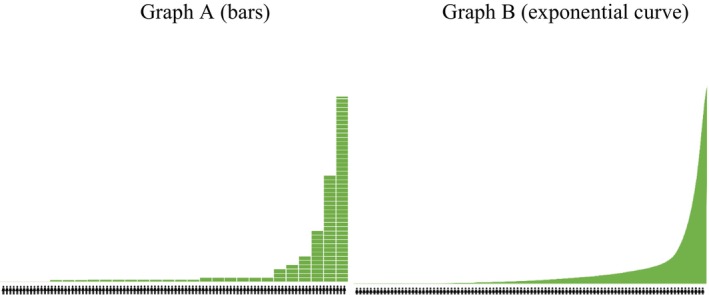
Graphs that were presented to the participants to represent the distribution of wealth in two different ways.

To facilitate the categorization of wealth‐based groups (Jetten et al., 2017), we used as wealth‐stimuli a graphical representation of the distribution of wealth in a fictional society based on data from the World Wealth Report (Shorrocks et al., 2020). The graph showed 100 individual figures, illustrating that the wealthiest 1% typically own between 25% and 40% of wealth, whereas the wealthiest 10% own between 55% and 75%. Two graphs (Figure 1) were used because of the possible influence of aesthetics. As a control, we accounted for the extent to which participants perceived economic inequality in each figure. We found no significant differences in individual respondents' responses on perceived inequality (see full description in Appendix S1).

Following this, participants were asked to delineate the wealth‐based groups by indicating where they believe group boundaries should be drawn. They also assigned labels to each group and described their main characteristics and traits. Notably, boundaries and labels varied across participants, reflecting individual conceptualizations of wealth‐based social divisions (see pictograms of participants' interactions with the graph in Appendix S1).

After the identification of the groups, we conducted a semi‐structured qualitative interview with open‐ended questions to explore how participants perceive and define wealth‐based groups. Participants were asked to describe the living conditions (e.g. possessions, consumption capacity, etc.), and outline daily behaviours and traits (e.g. habits, psychological traits, etc.) of the wealth‐based groups they identified. Specifically, participants answered the following questions: (1) ‘How could you group people in this distribution based on the wealth they have? How many groups could you create? Once this society is grouped into wealth‐based groups, what label would you give to each of these groups you have identified? Provide a label for each of these groups you have formed based on their wealth’; (2) ‘Think about the wealth of each group and imagine how they live, for example, where they live, what their house looks like if they have one, if they have their own vehicle, if they were able to go on vacation last summer, how they dress…in short, how each group lives? and (3) ‘Think about the personal characteristics of these groups, how they behave, what are their most outstanding traits, what type of music they listen to… in short, how would you describe these groups in terms of their personality and culture?’

All interviews were conducted via Google Meet, lasting between 9 and 28.02 min (M = 16.04, SD = 4.23). Informed consent was obtained from all participants before the study began. Additional exploratory quantitative data were collected via a Qualtrics survey (see Appendix S1).

#### Analysis plan

We used thematic content analysis to code participant's responses, combining deductive and inductive methods. This approach is useful for identifying patterns or themes within qualitative data through a reflective process of coding and interpretation (Braun & Clarke, 2022). We first developed general codes based on the research question to identify representations of wealth‐based groups, including labels and characteristics attributed to wealth‐based groups. Second, we inductively developed codes based on participants' responses, including traits, resources, culture, differences, similarities and social interactions (see complete coding framework in Appendix S1).

Three independent researchers coded the data. Researchers jointly reviewed and discussed a set of 30 interviews to establish shared criteria and ensure intersubjective agreement on how to use the coding schema. Then, each interview was independently coded in full by one researcher and then reviewed for accuracy and consistency by a second researcher. Any coding disagreements were resolved through discussion until consensus was reached.

We conducted two main analyses: a frequency analysis (Krippendorff, 2018) to examine the occurrence of each subcategory, identifying prevalent themes by counting each category and subcategory and a co‐occurrence analysis (Krippendorff, 2018) to explore the relationships between subcategories and their associations.

### Results

#### Frequency analysis

Representative categories include wealth‐based groups, traits, resources, culture, differences and similarities as well as contact and relations (Table 1). We analysed two categories in depth: wealth‐based groups and traits.

**TABLE 1:** Categorical framework.

Category (with definition)	Codes	Quotes examples	Frequency
**Wealth‐based groups** Groups defined by a set of individuals have in common the wealth they possess. Additionally, the people comprising each group share resources and culture than make them like each other and distinguish them from other groups	Poor Lower and working classes Middle classes Upper classes Rich and beyond	The first one would say homeless, the second one would be poor, a bit poor, the third one would be average, normal, the fourth one would be wealthy, and the fifth one would be millionaires or rich (P24) Very poor, poor, working class, upper middle class, rich or high class (P46)	1156
**Traits** The terms used to define personally, psychologically and culturally the groups of people categorized based on wealth. These terms can be positive, negative or neutral depending on the affective attitudes towards them	Positive Negative Neutral	I believe that the non‐wealthy are humble in the sense that, having nothing, they settle for very little. The normalised society, I would describe as ambitious, somewhere between humble and ambitious because some people may think, “Okay, what I have is enough, and I don't need much more,” but there's another part of society that wants to increase their wealth, climb the social ladder, and always wants more. The wealthy would be very ambitious, and I think they would be less generous. What's mine is mine, and I want my wealth (P25) The lower middle class and the poor are more honest because they have lived a different reality that the rich or the upper class have not experienced. So, I believe they have more camaraderie and empathy. Some of the upper‐class individuals, not all, will be a bit selfish or discriminate against the poor. The middle‐class group, I think, will have a bit of everything. Some will empathise more with the lower class because they see themselves reflected in them, while others will discriminate and try to pretend they have more (P59)	447

Abbreviation: (P), participant number.

##### Wealth‐based groups

Participants identified between two and seven groups based on wealth (Mdn = 4, SD = 1). Although the most common classification was three groups (35.6%), the majority (62.2%) of participants identified four or more, suggesting a more nuanced perception. The distribution was as follows: two groups (2.2%), three groups (35.6%), four groups (31.1%), five groups (25.6%), six groups (4.4%) and seven groups (1.1%). To balance complexity and interpretability without oversimplifying the data, we structured the classification into five groups, ensuring a symmetrical distribution and emphasizing the middle class.

Participants used a wide range of labels (113 codes in total), the most frequent being ‘poor’, ‘lower class’, ‘middle class’, ‘upper class’ or ‘rich’. We clustered the codes into five groups based on distinctions made by participants, semantic similarity and the sequence in which participants labelled them on the graph: (1) poor, (2) lower and working classes, (3) middle classes, (4) upper classes and (5) rich and beyond (see Table 2). Although some labels seem to be misplaced, they followed the participant's reasoning. For example, one person could have labelled the lowest class as ‘lower and working classes’ (Group 2) rather than ‘poor’ (Group 1), whereas millionaires could have been labelled as ‘upper classes’ (Group 4) rather than ‘rich and beyond’ (Group 5). Similarly, some participants placed ‘very low class’ in the Group 1, whereas others placed it in a slightly less disadvantaged category (Group 2); or ‘billionaires’ were generally positioned in a high group (Group 4), but not necessarily the highest (Group 5).

**TABLE 2:** Frequencies for the subcategories of the first main category: wealth‐based group.

Categories	Codes	Labels^a^	Frequency	Percentage (%)
Wealth‐based group	Poor	Poor, poverty, homeless, extreme poverty, poorest, poor, maximum poverty, super poor, needy…	322	27.85
	Lower and working classes	Lower class, working class, workers, working people, lumpen, insufficient wealth, very low economic level…	129	11.16
	Middle classes	Middle class, middle, upper‐middle class, normal, lower‐middle, intermediate, lower‐middle class…	303	26.21
	Upper classes	Upper class, high class, wealthy, privileged, excessive, powerful, most favoured class, high economic…	166	14.36
	Rich and beyond	Rich, very rich, millionaires, very high class, royalty, rich class, high bourgeoisie, fortunate, excessive…	236	20.42
Total			1156	100

^a^ Please refer to the complete code framework in Appendix S1.

In addition, some participants used terms to name the groups—for example, arrogant, whereas others used them to describe their associated traits, answering separate questions. Although the terms are the same, they indicate different things and were analysed separately.

We found that participants thought of wealth‐based groups in terms of ‘social class’, cited 725 times. This code encompassed references to consumption and material circumstances (e.g. ‘The middle class can afford to live well, but not luxuriously’, P80) or cultural interest (e.g. ‘…the dominant class has great artistic taste… they opt for beauty’, P64). This finding suggests that participants stratify society into social classes, reflecting differences and assumptions about economic relations between the classes. However, this view is based on descriptive criteria, with limited mention of power relations, as one participant noted: ‘The working class, well, I would say, obviously, works for the lower and upper bourgeoisie….’ (P10).

##### Traits

This category refers to the prominent traits to the previously mentioned groups. Individuals in the ‘poor’ group were described as kind, humble or happy; but also as distrustful, careless or conformist. Individuals in the ‘rich and beyond’ group were described as competent and happy; but also, as materialistic and arrogant. The so‐called ‘middle classes’ was vaguely described as a neutral group.

…the neutral group, who are not on one side or the other, they are the most common way of behaving… (p. 33)

To facilitate interpretation, we further categorized the traits according to their valence: positive and negative. This criterion was agreed upon by the researchers and evaluated by two external expert judges, with agreement measured by the Cohen's Kappa coefficient to ensure reliability (de Raadt et al., 2021).

The most used positive traits to describe the ‘poor’ group were ‘kind’, ‘happy’, ‘humble’, ‘solidary’, ‘empathic’ and ‘familiar’. However, negative traits such as ‘distrustful’, ‘desperate’, ‘conformist’, ‘bad’, ‘careless’ or ‘delinquent were also associated with this group. On the other hand, the ‘rich and beyond’ group was perceived as ‘materialistic’, ‘haughty’, ‘selfish’, ‘class unconsciousness’, ‘greedy’, ‘sad’, ‘unsociable’, ‘classist’ and ‘arrogant’. However, they were also positively described as ‘calm’, ‘happy’, ‘competent’ and ‘hardworking’. For a summary, see Appendix S1.^1^

#### Co‐occurrence analysis

A co‐occurrence analysis was conducted using the AND operator. This analysis examined the presence of multiple categories within the same recording unit, assessing the degree of association between positive or negative trait codes and wealth‐based group codes. We used Pearson's chi‐squared test to assess the independence between wealth‐based groups and trait valence, finding a significant association, *χ* ^2^ (4) = 56.35, *p* < .001, Cramer's *V* = 0.39, (OR) = 1.67, 95%, (CI) = [0.01, 0.85]. Bonferroni post‐hoc tests further explored associations between subcategories. Specifically, the ‘rich and beyond’ and ‘upper classes’ exhibited more negative traits and fewer positive traits, whereas the ‘poor group showed more positive traits and fewer negative traits. No significant associations were found for the ‘lower and working classes’ and ‘middle classes’ (Table 3).

**TABLE 3:** Bonferroni post‐hoc test of traits showing the standardized residuals between in brackets for testing independence for each group.

Wealth‐based groups	Traits		*p* Value
	Negative trait	Positive trait	
Poor	32 (−4.97)	64 (4.97)	<.001
Lower and working classes	16 (−2.18)	25 (−2.18)	.292
Middle classes	40 (−1.55)	44 (1.55)	>.999
Upper classes	43 (3.76)	12 (−3.76)	<.001
Rich and beyond	68 (5.14)	18 (−5.14)	<.001

*Note*: Standardized residuals in brackets.

### Discussion

We qualitatively explored the subjective categorization of wealth‐based groups by identifying the labels and traits people used to classify them. The qualitative analysis showed that the most used terms were ‘poor’, ‘lower class’, ‘working class’, ‘middle class’, ‘upper class’ and ‘rich’. These codes expanded into 113 codes (e.g. homeless, working class, normalized society, affluent class or super rich) which were further synthesized into five subcategories.

In the Spanish context, more than half of the participants did not perceive a tripartite structure (poor, middle class and upper class), suggesting a more nuanced view of social stratification. Moreover, although wealth was the most prominent criterion for categorization, the codes identified reflect both aspects of SES (economic position, education or occupation) and elements of social class (e.g. cultural practices consistent with Bourdieu's definitions). In this sense, the perception of wealth‐based groups among participants overlaps with broader notions of SES and social class and goes beyond a purely economic classification.

Although the term ‘social class’ appeared frequently, most of the categorization criteria were descriptive (e.g. based on income), with only a few explicit references to power relations, as emphasized in Marxist definitions. Thus, it suggests that, although people recognize social stratification, they tend to conceptualize it in terms of economic differences rather than structural inequalities.

Moreover, the descriptions of wealth‐based groups differed in the associated traits. Non‐wealthy groups were generally associated with positive traits, whereas wealthy groups were often attributed more negative traits. While traits can have positive or negative connotations, their content can be more nuanced and encompass ambivalence (Fiske et al., 2002). For example, while non‐wealthy groups may be seen negatively as conformist, suggesting a lack of ambition, they may also be characterized positively as humble, reflecting an ambivalent classism (Jordan et al., 2021; Sainz et al., 2021). Similarly, the rich are often described as immoral, but their materialism may be perceived positively, indicating competence due to ambition.

The qualitative nature of Study 1 introduces at least two limitations. First, the framing of the questions posed by the researchers may have guided participants' responses, even though participants responded freely from one question to the next. This limitation is common to both qualitative and quantitative research, as survey questions or open‐ended questions focus on aspects that do not necessarily reflect people's reasoning. Still, the questions we used during the interviews are useful to elicit participant's everyday experiences, as has been done in previous research (García‐Castro et al., 2022; García‐Sánchez et al., 2022). Second, although we aimed to reflect participant's reasoning through systematic coding, we acknowledge that researchers' subjectivity may have influenced data representation decisions. For example, we decided to group the wealth‐based groups into five clusters to resemble most people who identified more than four groups. This is a characteristic of qualitative research that, notwithstanding, provides the insights to unveil patterns of findings to deepen future research. In Study 2, we delve deeper into this association between wealth‐based groups and stereotypes.

## STUDY 2

In Study 2, we aimed to quantitatively analyse the association between wealth‐based groups and the valence of stereotypes attributed to them. Building on Study 1, we hypothesized that participants would use a higher percentage of negative traits to characterize wealthy groups (compared to non‐wealthy groups; H1) and a higher percentage of positive traits when evaluating non‐wealthy groups (compared to wealthy groups; H2), using the wealth‐based group subcategories generated from participants' labels (this study was pre‐registered, https://osf.io/25ecq/?view_only=3db493cdddd1432e887796ac0aecb241).

Additionally, we explored the stereotype content and structure of wealth‐based groups by using psychometric networks. This approach is useful to understand the structure of stereotypes (Sayans‐Jiménez et al., 2019), attitudes and behaviours (Chambon et al., 2022; Dalege, Borsboom, van Harreveld, & van der Maas, 2017).

### Method

#### Participants

We conducted an a priori power analysis for chi‐squared using the ‘pwr’ package in RStudio (Champely et al., 2017). To detect a medium effect size of *w* = 0.30 (*df* = 2) with 80% power and an alpha error of 0.05, a sample size of 174 was required. Therefore, we pre‐registered a minimum sample size of 200 participants.

We recruited 258 participants but excluded seven due to language proficiency or age pre‐register criteria. The final sample consisted of 251 participants (181 women, 63 men, 1 non‐binary and 3 ‘prefer not to say’), aged 18–66 years (M = 24.744, SD = 8.97). Participants identified on average as centre‐left on economic (M = 3.25, SD = 1.49) and social issues (M = 2.63, SD = 1.30) on a scale ranging from 1 (radical left) to 7 (radical right). They reported a mean subjective SES of 5.98 (SD = 1.30) on a scale of 1–10.

#### Procedure

The sample was recruited through incidental sampling via the University of Granada mailing list, reaching undergraduate and graduate students as well as faculty and staff members. The study was conducted via a Qualtrics web link, and participants were incentivized with a raffle for 50 euros.

**FIGURE 2:**
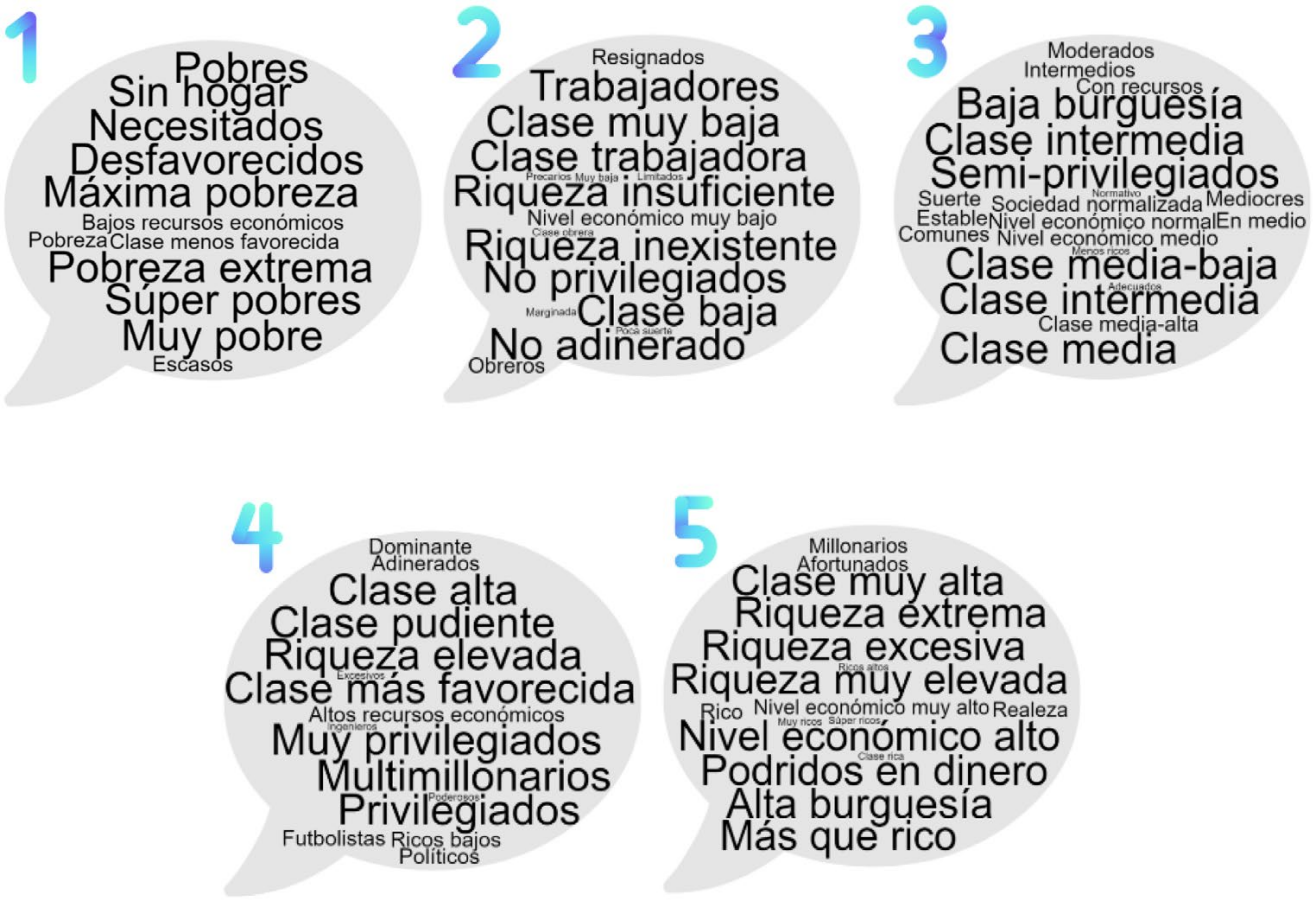
Word clouds representing the codes contained in the subcategories.

Participants were informed that ‘social class’^2^ would be used, defined as ‘The population of a society can be described as composed of several social classes. A social class is a group of people with similar levels of wealth, occupation, and education’. They were shown five‐word clouds (see Figure 2; Appendix S1) containing common terms for wealth‐based groups drawn from participants' labels identified in the subcategories of Study 1: (1) poor, (2) lower and working classes, (3) middle classes, (4) upper classes and (5) rich and beyond. Participants did not see the terms we used to label each wealth‐base group but created their own terms for each word cloud. Thus, when they responded to the measures, they saw both the word cloud and the self‐created term.

#### Analysis plan

To test our main hypotheses, we used a Pearson chi‐squared analysis to compare the percentages of negative traits assigned to wealthy versus non‐wealthy groups and positive traits assigned to non‐wealthy versus wealthy groups. This analysis was performed for each trait across the five wealth‐based groups.

We carried out the ‘chisq.test’ function and the ‘chisq.posthoc.test’ package in R (R Core Team, 2017), through the RStudio interface. A proportions table was created with the subcategories (poor, low‐ and working‐classes, middle‐classes, upper‐classes and rich and beyond) as rows and the trait (e.g. negative traits as ‘Selfish’ and positive traits as ‘Kind’) as columns, using the ‘matrix’ function. This table represents the percentage of participants assigning each trait to the groups, indicating whether the trait was characteristic of the group or not. For a robustness check, we performed two meta‐analyses with random effects to assess consistency in all trait associations with each wealth‐based group, using the DerSimonian‐Laird estimator (DerSimonian & Laird, 1986) and the ‘metafor’ package (Viechtbauer, 2010) in RStudio.

Additionally, we employed the Causal Attitude Network (CAN) model (Dalege et al., 2016) to map the stereotype content and structure of wealth‐based groups to test the connections between variables and identify the underlying structure of those associations. Considering that the meaning of some stereotypes depends on the valence of neighbour attributes (e.g. strong‐violent vs. strong‐brave), using a network approach allows us to extend the valence of individual stereotypes to identify clusters that capture more complex and accurate representations of wealth‐based groups. Therefore, we contribute to the theory of stereotypes as complex networks by showing that wealth‐based representations are not just the sum of single attributes, but the combination of all of them (Sánchez‐Rodríguez et al., 2023; Sayans‐Jiménez et al., 2019). The network comprises nodes (variables) and edges (semi‐partial correlations). Following the literature (Dalege, Borsboom, van Harreveld, Waldorp, & van der Maas, 2017), we used the Ising model (with regularized method) for binary data (1 = marked; 0 = unmarked) to estimate the association. This model computes associations between all node pairs while controlling for the influence of the remaining nodes.

#### Measures

##### Traits

Participants were shown 47 traits that may characterize each wealth‐based group (28 negative, such as arrogant or careless; and 19 positive, such as humble or competitive). Participants did not know whether the traits were classified as positive or negative and could select the traits that were characteristic of each group. These traits were obtained from Study 1. They had the option to select multiple wealth‐based groups for the same trait or choose only one wealth‐based group, as well as not to ascribe any trait to any group.

##### Feeling thermometer

Participants were asked to rate each word cloud on a scale from 0 (colder, less favourable) to 100 (warmer, more favourable) based on how they felt about the wealth‐based group represented by the set of words.

##### Sociodemographic

Finally, some sociodemographic data were requested: Subjective SES (Adler et al., 2000), objective SES, educational level, political ideology focused on economic and social issues with two items (‘Radical left’, ‘Center left’, ‘Center’, ‘Center right’, ‘Right’ and ‘Radical right’), gender, age, language and nationality.

### Results

#### Preliminary results

##### Feelings towards wealth‐based groups: Warm (or favourable) and cold (or unfavourable)

The repeated measures ANOVA revealed a main effect of wealth‐based group, *F* (1.84, 460.82) = 192.85, *p* < .001, *η* ^2^ = 0.43, indicating significant differences in participants' feelings across groups. Notably, the ‘rich and beyond’ group (M = 21.71, SD = 1.62) was perceived as less favourable—less warm—compared to both the ‘poor’ group (M = 57.36, SD = 1.86) and ‘lower and working classes’ group (M = 67.76, SD = 1.67), with *p* < .001 in both contrasts. Similarly, the ‘upper classes’ group (M = 30.49, SD = 1.61) was perceived as less favourable compared to both the ‘poor’ and ‘lower and working classes’ groups. Conversely, the ‘middle classes’ group (M = 63.04; SD = 1.37) was perceived more favourably than the ‘upper classes’ and ‘rich and beyond’ groups (all *ps* < .001), but showed no significant difference compared to the ‘lower and working classes’ group (*p* = .085) or ‘poor’ group (*p* = .058). Additionally, participants perceived the ‘rich’ group less favourable than the ‘upper class’ group, while the ‘lower and working classes’ group was viewed more favourably than the ‘poor’ group (all *ps* < .001).

#### Main results

##### Perception of wealth‐based groups in terms of negative traits

**FIGURE 3:**
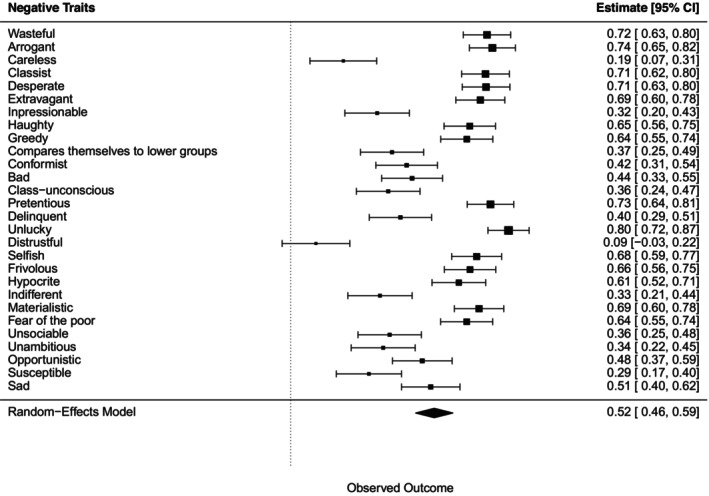
Meta‐analysis of the effects of Cramer's *V* for each association between negative traits and the groups.

A chi‐squared test for each negative trait, followed by a meta‐analysis, showed all associations with wealth‐based groups were statistically significant, except for the negative trait ‘distrustful’ (Figure 3).

Bonferroni post‐hoc tests revealed that 19 traits out of 21 negative traits were associated with the ‘rich and beyond’ group (e.g. selfish, haughty, class‐unconscious, materialistic, arrogant, greedy, pretentious, unsociable, bad, indifferent, opportunistic, delinquent, wasteful, classist, extravagant, hypocrite, fear of the poor, frivolous), and 14 were associated to the ‘upper classes’ group (e.g. haughty, selfish, materialistic, arrogant, greedy, pretentious, indifferent, opportunistic, wasteful, classist, extravagant, hypocrite, fear of the poor, frivolous). Meanwhile, six traits were associated with the ‘poor’ group (e.g. sad, desperate, delinquent, unambitious, unlucky, susceptible) and similar to ‘lower and working classes’ group. In the case of the ‘middle classes’ group, only two traits are identified (e.g. conformist and compare themselves to lower groups).

##### Perception of wealth‐based groups in terms of positive traits

**FIGURE 4:**
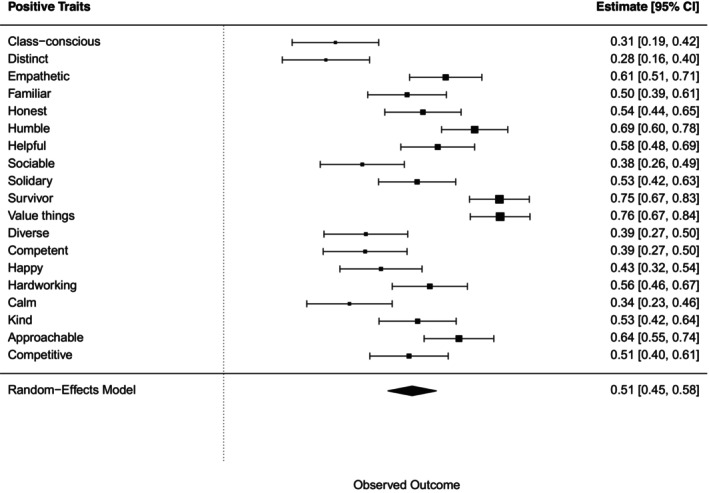
Meta‐analysis of the effects of Cramer's *V* for each association between positive traits and the groups.

A chi‐squared test for each positive trait, followed by a meta‐analysis, showed all associations with wealth‐based groups were statistically significant (see Figure 4).

Bonferroni post‐hoc tests showed that ‘lower and working classes’ was associated with 12 of 20 positive traits (e.g. humble, kind, value things, solidary, empathetic, hardworking, class‐conscious, familiar, approachable, survivor, helpful, honest). The ‘poor’ group was associated with four positive traits (e.g. humble, value things, survivor, helpful) and the ‘rich and beyond’ group was characterized by two positive traits (e.g. distinct, competitive). In the case of the ‘middle classes’ group, 17 traits were identified. A detailed summary of these results can be found in the Appendix S1.

##### Psychometric network analysis to map wealth‐based stereotypes

**FIGURE 5:**
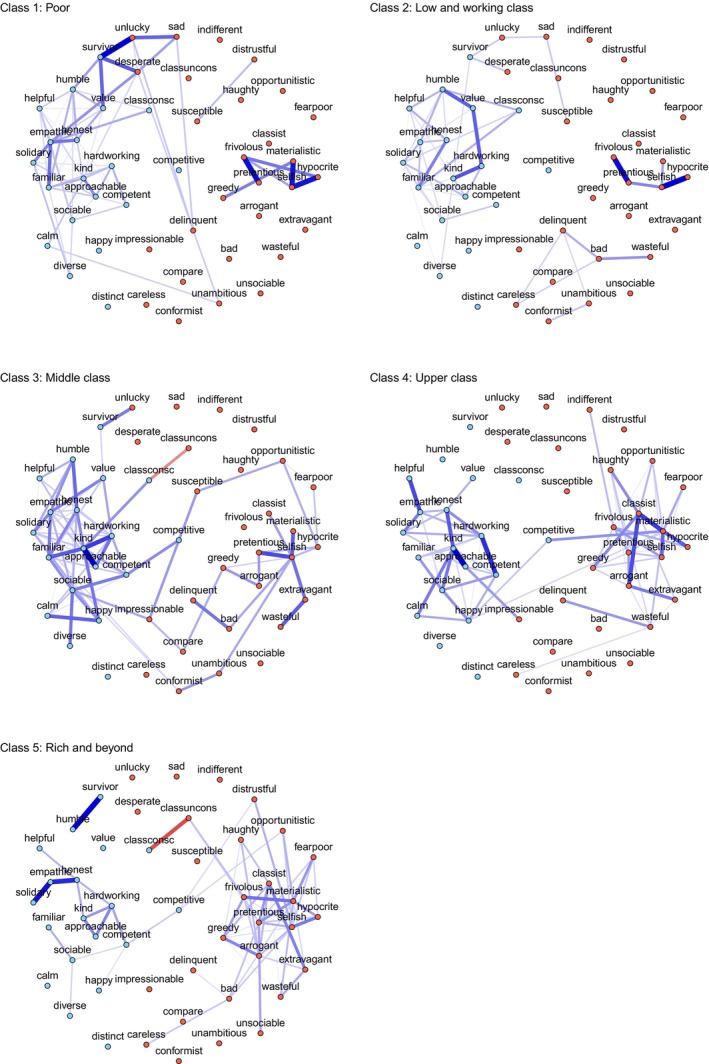
Psychometric network analysis of wealth‐based groups: poor, low and working classes, middle class, upper class and rich and beyond.

Figure 5 illustrates the network of wealth‐based group stereotypes. The nodes were coloured based on the valence of the traits (i.e. positive in light blue, and negative in light red). The thickness of the links between nodes indicates the strength of the association between nodes, estimated from the Ising's model, and the link colour indicates whether the association is positive (blue) or negative (red). The links between nodes indicate the unique association between two variables after controlling for all other variables in the network (partial correlations). Non‐statistically significant relationships are omitted from this network to facilitate readability. We used the Fruchtermon‐Reingold algorithm to visualize the network by averaging the layout parameters of the five networks.

In addition to the association of positive traits with non‐wealthy groups and negative traits with wealthy groups, psychometric networks provide additional insight into the structure and content wealth‐based groups stereotypes. The structure of stereotypes shows that positive traits were more concentrated and interconnected among the ‘poor’ and ‘lower and working classes’ groups compared to the ‘upper classes’ and ‘rich and beyond’ groups. In contrast, negative stereotypes were more varied and more densely interconnected in the ‘upper classes’ and ‘rich and beyond’ groups compared to the ‘poor’ and ‘lower and working classes’ groups.

The content of stereotypes also reveal which traits are more central to each group. Regarding the ‘poor’ group, the stereotype revolves around central nodes such as empathetic, familiar, survivor, kind and humble. Negative traits, such as frivolous or selfish, are present but show weaker interconnections compared to the positive traits. Notably, we observed associations between positive and negative traits, highlighting the ambivalence of wealth‐based group stereotypes. For example, traits such as empathetic, familiar, and survivor are connected to desperate and unlucky. This pattern suggests a perception of the ‘poor’ as having a positive moral character, while also acknowledging the structural factors of poverty and expressing empathy for their experiences.

In contrast, the highest centrality nodes for the ‘rich and beyond’ group were predominantly negative, such as materialistic, pretentious, selfish, arrogant and classist. Positive traits, such as empathetic, honest and hardworking, show much lower centrality and density in terms of their interconnections. The clusters of positive and negative traits were not associated, indicating two different types of representation: one related to materialistic and classist individuals (e.g. wealthy that look down upon others) and other about hardworking and empathetic individuals (e.g. self‐made people who moved upward in the social ladder).

The other ‘lower and working classes’, ‘middle classes’ and ‘upper classes’ were somewhat in between the representations of the ‘poor’ and the ‘rich and beyond’. The ‘working class’ showed a denser connection of both positive and negative traits compared to the ‘poor’ group. The most central nodes for this group included ambivalent traits, such as selfish, humble, familiar, hardworking, pretentious, value, solidary, empathetic and honest. The ‘middle classes’ group shows central nodes such as kind, selfish, sociable, hard‐working, empathetic, family‐oriented, honest, caring, competent and happy. The structure reflects positive traits forming denser interconnections and fewer negative traits, showing more isolated clusters of nodes. Finally, the ‘upper classes’ shows a denser structure of nodes reflecting negative traits, such as materialistic, classist and arrogant, with less dense nodes interconnections of positive traits, such as hard‐working, competent and empathetic.

### Discussion

In Study 2, we extended the qualitative results of Study 1 in terms of trait use. Corroborating our Hypotheses 1 and 2, participants attributed a higher percentage of negative traits to characterize wealthy groups (compared to non‐wealthy groups) and a higher percentage of positive traits when evaluating non‐wealthy groups (compared to wealthy groups). However, the ‘poor’ were not as positively stereotyped as the ‘low‐ and working‐class’ or ‘middle class’ groups.

Non‐wealthy groups (‘poor’ and ‘lower and working classes’) and the ‘middle‐class’ were perceived more favourably compared to wealthy groups (‘upper classes’ and ‘rich and beyond’). It is important to note that not all non‐wealthy groups were perceived equally; the ‘lower and working classes’ group were viewed more positively than the ‘poor’. Similarly, within the wealthy groups, the ‘rich’ were evaluated more negatively than the ‘upper classes’ group. This reflects the importance of considering a broader framework of possible intermediate groups that lay perceptions reflect.

Regarding the networks, the ‘poor’ and ‘lower and working classes’ groups were seen with a mix of positive human qualities, reflecting warmth and sociability (empathetic, familiar and kind) and negative features that shape difficulties, which can signal their resilience as well as systemic structural symptoms (survivor, desperate and unlucky). The ‘middle classes’ had a more homogenous and positive image, with a combination of effort, sociability and competence. The ‘upper classes’ was ambivalent, combining admiration with stereotypes of elitism. Finally, the ‘rich and beyond’ was predominantly perceived negatively, with moral aspects associated with the way they acquire status and power, as well as social disconnection. Taken together, these results show that the perception of wealth‐based groups does not simply reduce to being ‘positive’ or ‘negative’. Instead, it is structured around networks of meanings that reveal how each group is conceptualized in terms of its values, behaviours or perceived morality.

The network analysis advances our understanding of wealth‐based stereotypes in two ways. First, it conveys the idea that stereotypes are formed by a combination of different traits, forming clusters that better capture a broader image than looking at individual traits (Sánchez‐Rodríguez et al., 2023; Sayans‐Jiménez et al., 2019). Thus, we can observe tighter relationships among the middle‐class and more diverse connections among groups at the extreme. Second, the stereotype network allows us to recognize that the valence of stereotypes is not systematically unipolar, as positive and negative traits coexist to create compensatory stereotypes. For instance, wealth‐based stereotypes combine positive and negative attributes, such as ‘poor but happy’ for the disadvantaged and ‘competitive but distrustful’ for the advantaged (Kay & Jost, 2003). Indeed, the most advantageous combination of the highest social groups yields provoking results along these lines, with two consistent clusters of negative and positive traits coexisting in the five groups. For the poor, the highest density of relationships between traits is in the cluster of positive traits, whereas for the rich, the relationships are denser in the negative cluster. However, positive and negative clusters are connected for both groups, indicating the ambivalence of stereotypes. Beyond the trait‐by‐trait analysis, the results show how positive and negative traits dynamically construct the content of the stereotype.

It is possible that the word clouds used in Study 2 included terms with inherent valence, which could bias participants' responses (e.g., cheerful, resigned or rotten with money). These clouds were constructed from the codes that emerged spontaneously in the previous qualitative study. Consequently, participants were not equally exposed to positive and negative labels, which may have influenced the valence of the stereotypes attributed to each group. In terms of traits, we excluded characteristics such as being highly educated or caring about money, as these may be descriptive statements rather than forming the core of a stereotype. Since both the group codes and the traits emerged directly from the participants' responses, all the words used may have a degree of valence that is difficult to completely neutralize. However, to mitigate this potential bias, Study 3 was designed to test these results under conditions that minimized it, using wealth‐neutral stimuli and excluding the traits from the analyses.

## STUDY 3

Study 3 sought to confirm the previous findings (Study 2) by analysing the percentage of traits used for each of the five groups. We pre‐registered two main hypotheses:

Hypothesis 1 *Non‐wealthy groups are expected to be evaluated more positively than wealthier groups. Specifically, groups 1 and 2 will be evaluated more favourably than groups 4 and 5*.

Hypothesis 2 *Participants are expected to use a higher percentage of negative traits for wealthy groups 4 and 5 (compared to non‐wealthy groups 1 and 2) and a higher percentage of positive traits for non‐wealthy groups 1 and 2 (compared to wealthier groups 4 and 5)* (https://osf.io/8f3pr/?view_only=deb5b9d7f35d410e96df7ce86eab6065).

### Method

#### Participants

We performed an a priori power analysis using the ‘pwr’ package for RStudio (Champely et al., 2017). First, for the repeated measures ANOVA, we determined that a sample size of 36 was needed to detect a medium effect size of *f* = 0.30 with 90% power and an alpha error of 0.05. Second, for a paired samples *t*‐test, the sample size required to detect a medium effect size of *d* = 0.30 with 90% power and alpha error (Bonferroni adjusted for 10 comparisons) was 189 observations. Finally, for a chi‐squared test, a sample size of 174 was required to detect a medium effect size of *w* = 0.30 (*df* = 2) with 80% power and an alpha error of 0.05. Therefore, we pre‐registered a minimum sample size of 200 participants.

A total of 200 participants took part in Study 3. All met the pre‐registered criteria of having a ‘native’ or ‘advanced’ level of Spanish and being 18 years or older. We excluded 10 participants who misunderstood the procedure, specifically those who misinterpreted the least wealthy groups as the wealthiest, and vice versa (see procedure). The final sample comprised 190 participants (61 women, 120 men, 8 non‐binary and 1 ‘other), aged 19–80 years (M = 33.30, SD = 10.70). Politically, participants identified themselves on average as centre‐left (M = 38.70, SD = 22.80) on a scale from 1 (radical left) to 100 (radical right). They reported a mean subjective SES of 5.72 (SD = 1.42) on a scale of 1–10.

#### Procedure

The sample was collected via the *Prolific* platform, and the study was administered through a *Qualtrics* web link, with participants compensated at a rate of £7.53 per hour.

**FIGURE 6:**
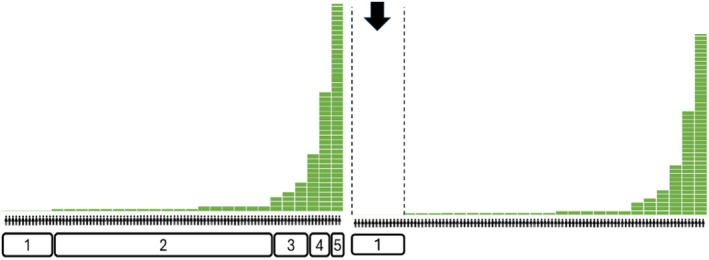
Graph of wealth distribution segmented by five wealth‐based groups (Step 1 on the left) and example of Group 1 signalling (Step 2 on the right).

Study 3 closely mirrored Study 2, however, we avoid using word clouds included terms that might convey implicit valence of social class (e.g. ‘needy’, ‘rotten with money’) or terms that did not fit neatly into the proposed group categories (e.g. ‘football player’). Instead, we used more neutral stimuli. Specifically, participants interacted with a wealth graph segmented into five groups (see Figure 6, see Appendix S1 for more examples). They were informed that ‘The graph shows how wealth is distributed, from those with the least wealth to those with the most, in ascending order’ and ‘The graph can be divided into different groups according to their wealth. For example, it can be segmented into 5 groups, ranging from Group 1 (the people with the least wealth) to Group 5 (the people with the most wealth), through Group 2, Group 3 and Group 4 in ascending order of wealth’.

Participants were asked to identify and name each wealth group to confirm their understanding of the procedure of the graph. We excluded 10 participants due to incorrect use of terms. That is, in order, they labelled the first group (the lowest wealth group) with a wealthy group label, and so on consecutively for groups 2, 3, 4 and 5. Finally, participants responded to the measures provided.

#### Analysis plan

First, to test Hypothesis 1, we performed repeated measures ANOVA using the statistical software Jamovi Version 2.3. Second, to test Hypothesis 2, we followed the same analysis plan as in Study 2 (i.e. chi‐squared tests, mini meta‐analysis encompassing the effect of each trait and post‐hoc comparisons, RStudio).

#### Measures

We used the same measures as in Study 2 (i.e. Feeling thermometer, traits, sociodemographic).

### Results

#### Feelings towards wealth‐based groups: Warm (favourable) and cold (unfavourable)

The repeated measures ANOVA revealed a main effect of wealth‐based group, *F* (4,189) = 93.1, *p* < .001, *η* ^2^ = 0.26. Significant differences were found with the ‘rich and beyond’ group (M = 30, SD = 28.10), which was perceived as less favourable compared to the ‘poor’ group (M = 60.50, SD = 24.40) and the ‘lower and working classes’ group (M = 67.30, SD = 21), both *ps* < .001. The ‘upper classes’ group (M = 46.50, SD = 21.90) was also perceived as less favourable than ‘poor’ and ‘lower and working class’ groups. However, the ‘middle class’ group (M = 62.60; SD = 18.40) was perceived more favourably than the ‘upper classes’ and ‘rich’ groups (all *ps* < .001), but similarly to the ‘poor’ and ‘lower and working classes’ groups (*p* = .999; *p* = 060). Additionally, participants perceived the ‘rich’ group as less favourable than the ‘upperclasses’ and the ‘lower and working class’ group more favourably than the ‘poor’ group (all *p* < .001).

#### Perception of wealth‐based groups in terms of negative traits

**FIGURE 7:**
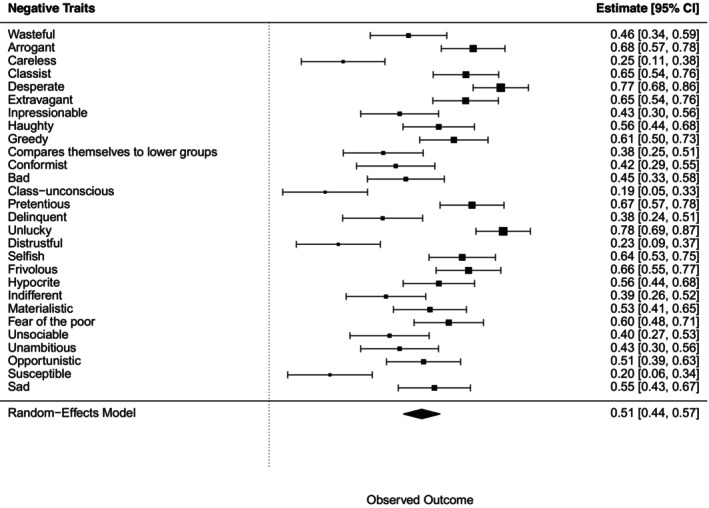
Meta‐analysis of the effects of Cramer's *V* for each association between negative traits and the groups.

A chi‐squared test for each negative trait, followed by a meta‐analysis, showed all associations with wealth‐based groups were statistically significant (Figure 7).

Bonferroni post‐hoc tests revealed that19 out of 29 negative traits were associated with ‘rich’ group (e.g. haughty, selfish, class‐unconscious, materialistic, arrogant, greedy, pretentious, unsociable, bad, indifferent, opportunistic, delinquent, wasteful, classist, extravagant, distrustful, frivolous, hypocrite, fear of the poor) while seven traits were associated with ‘poor’ group (e.g. sad, careless, delinquent, unambitious, impressionable, unlucky, susceptible) and also with ‘lower and working classes’ group. Only three negative traits were associated with the ‘middle class’ group (e.g. conformist, impressionable, compared themselves to lower groups).

#### Perception of wealth‐based groups in terms of positive traits

**FIGURE 8:**
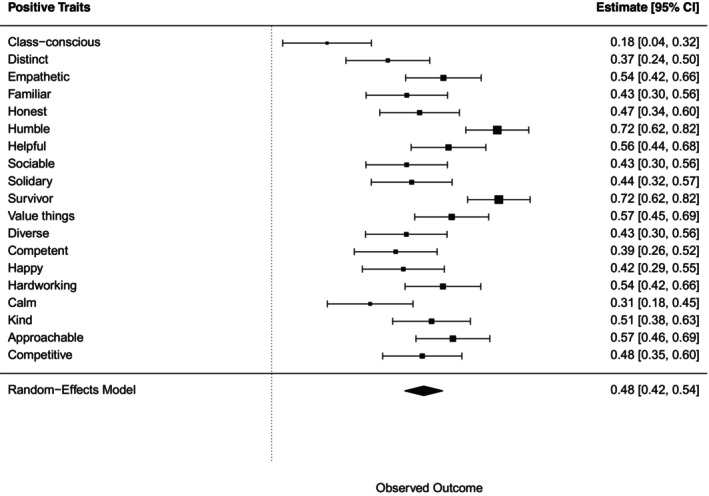
Meta‐analysis of the effects of Cramer's *V* for each association between positive traits and the groups.

A chi‐squared test for each positive trait, followed by a meta‐analysis, indicated a statistically significant association between positive traits and wealth‐based group categorization (Figure 8).

Bonferroni post‐hoc tests showed that ‘lower and working classes’ group was associated with 14 of 29 positive traits (i.e. humble, kind, value things, solidary, empathetic, hardworking, class‐conscious, familiar, approachable, survivor, sociable, helpful, honest, diverse). ‘Poor’ group was associated with five positive traits (i.e. humble, value things, empathetic, survivor, helpful) ‘middle class’ group with 16 (i.e. kind, happy, value things, solidary, empathetic, hardworking, distinct, familiar, competent, approachable, sociable, helpful, competitive, honest, calm, diverse), and ‘rich’ group with two (e.g. distinct, competitive). Refer to the Appendix S1 for a summary of these results.

### Discussion

In Study 3, we corroborated the findings of Study 2 using more neutral stimuli. Regarding Hypothesis 1, we found that lower wealth groups (poor and lower and working classes; Groups 1 and 2) were perceived more favourably than higher wealth groups (upper classes and rich and beyond; Groups 4 and 5). The ‘lower and working classes’ and ‘middle classes’ (Groups 2 and 3) were the most favourably perceived compared to the others, with no significant differences between them. As in Study 2, the ‘lower and working classes’ group was perceived more positively than the ‘poor’ group. Similarly, within the wealthier groups, the ‘rich’ group was evaluated more negatively than the ‘upper classes’ group. A similar pattern was observed in stereotyping. In line with Hypothesis 2, most negative traits were associated with wealthier groups (upper classes and rich and beyond; Groups 4 and 5), whereas positive traits were linked to lower wealth groups (Groups 1 and 2). Specifically, the ‘lower and working classes’ group was more positively perceived, whereas the ‘poor’ group did not receive such positive stereotypes, as in Study 2. Thus, wealth‐based stereotypes remained consistent, regardless of whether participants' own labels (Study 2) or neutral stimuli (Study 3) were used.

In line with these results, we tested, in a separate study using a representative data set from Spain (see Appendix S1), the association between favourable attitudes towards the poor and working‐class groups and unfavourable attitudes towards the rich. The results revealed that the rich were perceived less favourably than the poor and working class, with the latter being viewed more favourably than the poor group (for more details, see the Appendix S1).

## GENERAL DISCUSSION

This research explored lay perceptions of social class by examining how individuals categorize wealth‐based groups (Study 1), focusing on the content and structure of stereotypes attributed to these groups (Studies 2 and 3). Our qualitative approach provided a subjective perspective on participants' views of social stratification in Spain, avoiding the constraints of predefined categories. Study 1 found that 56.6% of participants identified four to five wealth‐based categories. To preserve the diversity of these lay perceptions, we grouped according to the data these categories as poor, lower and working classes, middle classes, upper classes and rich and beyond. This categorization formed the empirical basis for Studies 2 and 3, which further showed that these groups were evaluated and stereotyped differently. Non‐wealthy groups—especially the lower and working classes—were evaluated more favourably and were associated with more positive traits and fewer negative traits than the wealthier groups. Furthermore, psychometric network analyses revealed ambivalent stereotypes, with positive and negative traits associated with each group.

Research on stereotype content has shown that attitudes towards wealth‐based groups tend to be negative towards the poor, mostly positive towards the middle class and ambivalent towards the rich (Cuddy et al., 2008; Fiske et al., 2002; Fiske & Markus, 2012). However, our findings reveal a different pattern. Non‐wealthy groups (the poor and the lower and working classes) were perceived more favourably than the wealthy groups (the upper classes and the rich and beyond). Similarly, non‐wealthy groups were associated with fewer negative traits and more positive traits compared to wealthy groups. These findings remain consistent across Studies 2 and 3, confirming our hypothesis. In both empirical tests (the affective thermometer and the trait meta‐analyses), the lower and working classes were evaluated more positively. This suggests that wealth‐based perception and stereotyping in the Spanish context differs from existing models, which primarily associate disadvantaged and working‐class individuals with negative traits while attributing positive traits to the wealthy. This divergence may reflect cultural, political, and economic factors specific to Span. Spain was severely impacted by the 2008 economic crisis, which resulted in high unemployment rates, mass evictions, and income loss for most households, while large corporations were bailed out and political corruption scandals emerged (Carballo‐Cruz, 2011; Malo, 2015; Royo, 2020). The economic repercussions are still felt today, sustaining a narrative that favours hardworking low socio‐economic groups while criticizing the wealthy. Further research could explore whether and how economic shocks shape people's stereotypes of social classes.

Additionally, psychometric networks contribute to advancing our understanding of social class stereotypes by mapping both the content and structure of attributes associated with each social group. Rather than analysing individual traits as isolated entities, the meaning of stereotypes depends on the diversity and strength of their interconnections. For example, in the lower social class group, ‘survivor’ is linked to ‘unlucky’ and ‘desperate’, reflecting structural factors associated with poverty. In contrast, in the higher social class group, ‘survivor’ is only connected to ‘humble’, potentially alluding to notions of upward social mobility. Moreover, the strength of these associations suggests that ‘survivor’ is a more central characteristic in the lower social class group than in the working and upper classes. Previous research in line with the SCM (Durante et al., 2013, 2017; Fiske et al., 2002) has shown that the rich are perceived as cold (e.g. frivolous) but competent (e.g. competitive), while the poor are seen as warm (e.g. humble) but incompetent (e.g. unambitious). The psychometric network approach in Study 2 expands on this by suggesting that stereotypes do not operate as a mere sum of positive or negative traits, but as a combination of interconnected attributes (Sánchez‐Rodríguez et al., 2023; Sayans‐Jiménez et al., 2019). Consistent with our hypotheses, positive trait connections were denser for the poor, whereas negative trait connections were denser for the rich.

These results offer valuable insights into the complex dynamics of wealth‐based stereotypes and their potential societal functions. Perceptions of the poor align with the horizontal dimension of the social evaluation model, particularly assertiveness (Abele et al., 2021). This may serve dual functions: compensating for negative stereotypes and justifying the system (Kay et al., 2007). In contrast, the rich group tends to be evaluated primarily in terms of morality (e.g. arrogant, classist or frivolous). These findings support the notion that the rich are perceived as less kind and moral than the poor (e.g. Moreno‐Bella et al., 2019). Moreover, perceptions of immorality among the rich may stem from the belief that their wealth was acquired illegitimately (Sussman et al., 2014; Tao et al., 2016). While the perceived lack of competence of the poor—especially in persistent poverty—can reduce support for social protection policies (Alcañiz‐Colomer et al., 2023), perceiving the rich as immoral can boost support for progressive taxation (Tanjitpiyanond et al., 2022). Thus, stereotypes simultaneously serve to legitimize social hierarchies (e.g. justify status differences, Kay & Friesen, 2011) and challenge them when the dominant group is perceived as immoral.

One of the key findings in this research is the relatively more positive perception of the lower and working classes, as well as the middle classes, compared to the poor. Large‐scale surveys with representative samples indicate a general tendency for individuals to identify with these two social class groups: the middle‐class (US) and the working‐class (UK) (Evans & Mellon, 2016; Marsden et al., 2020). This identification fosters a heuristic in which people adhere to a ‘middle‐class’ consensus that blurs the boundaries between wealth‐based groups (Evans & Kelley, 2004). Moreover, as with other aspects of identity, individuals attribute meaning and value to their social classes, developing a sense of belonging and placing a more positive value on their ingroup (Destin et al., 2017; Easterbrook et al., 2020; Spears, 2011). Indeed, individuals tend to stereotype their own group less harshly on dimensions that are generally perceived negatively (Elenbaas et al., 2024). Given that these group identities are normatively extended, it is consistent that they are also stereotyped more positively compared to other groups in our research.

Overall, our results enhance understanding of wealth‐based categorization and stereotypes, along with their political implications. Study 1's qualitative approach enhances this by highlighting additional structural features in the representation of wealth‐based groups beyond material terms, such as cultural habits (Becker et al., 2017). While wealth shapes intergroup boundaries (Jetten et al., 2017, 2021), our analysis suggests wealth‐based perceptions align more with income‐based class frameworks (e.g. SES; Kraus & Stephens, 2012) than with Marxist analyses of economic power relations (e.g. Bradley, 2014; Wright, 2015). This distinction reflects how lay perceptions often overlook the power‐based aspects of class stratification that drive class consciousness and collective action (e.g. perceptions of injustice, Klandermans, 2014). Future research could explore how Marxist versus descriptive wealth labels (e.g. SES) influence views on economic inequality and class stereotypes.

This work has some limitations that limit the generalizability of our findings. First, the samples are predominantly WEIRD (White, educated, industrialized, rich, democratic). In addition, the sample in Study 1 was mostly university students, a limitation that we overcame by using other samples in Studies 2 and 3. Although the interviews were conducted in a neutral manner, the questions prompted participants to think about everyday experiences, such as possessions, consumption habits, lifestyles, and tastes (García‐Castro et al., 2022; García‐Sánchez et al., 2022). Although this may have directed some responses, participants had the opportunity to elaborate on their responses and make sense of the wealth‐based groups using references that were meaningful for them. Overall, replications in other cultural contexts and the use of representative samples would be important to increase the generalizability of these findings.

In sum, this research shows that laypeople's perceptions of social classes challenge traditional frameworks by incorporating subjective dimensions. Wealthier groups were generally perceived more negatively than non‐wealthy groups, though not uniformly, reflecting ambivalent stereotyping. These findings call for a broader approach to social class research, one that accounts for the nuanced ways in which people stratify society and the context in which these perceptions are formed.

## AUTHOR CONTRIBUTIONS

**Ángel del Fresno‐Díaz:** Conceptualization; methodology; writing – original draft; writing – review and editing; visualization; investigation; software; formal analysis; data curation; project administration. **Efraín García‐Sánchez:** Conceptualization; methodology; software; data curation; writing – original draft; writing – review and editing; visualization; investigation. **Elena Padial‐Rojas:** Data curation; formal analysis; methodology; software; visualization; investigation. **Guillermo B. Willis:** Methodology; funding acquisition; writing – original draft; writing – review and editing; visualization; resources; investigation; conceptualization; supervision; project administration. **Soledad de Lemus:** Supervision; resources; project administration; methodology; visualization; writing – review and editing; writing – original draft; funding acquisition; investigation; conceptualization.

## CONFLICT OF INTEREST STATEMENT

No potential conflict of interest was reported by the author(s).

## Supporting information


Appendix S1

## Data Availability

The preregistration for Studies 1, 2 and 3 are openly available upon the Open Science Framework (OSF) website: (Study 1: https://osf.io/9wzmj/?view_only=8c5e62f11f774053b80b35e124ec101f; Study 2: https://osf.io/25ecq/?view_only=3db493cdddd1432e887796ac0aecb241; Study 3: https://osf.io/8f3pr/?view_only=deb5b9d7f35d410e96df7ce86eab6065). Qualitative content, questionnaires and R code used in Studies 1, 2 and 3 are available at https://osf.io/549mq/?view_only=68bbb37826064539813a6f5f4f371c6d.

## Footnotes

^1^
Some participants attributed traits such as highly educated, concern about wealth or with economically difficulties. This may reflect how these traits are used to ascribe stereotypes in the Spanish context. Since these traits can be interpreted as descriptive rather than strictly stereotypical, we have decided to exclude them from the main text and the analyses that involve Studies 2 and 3.

^2^
This research focuses on wealth‐based categorization but will be influenced by the perception of social classes. Therefore, in this case, we used social class as an anchor to facilitate the understanding of the task, as it is a more familiar concept.

## References

[bjso70003-bib-0001] Abele, A. E. , Ellemers, N. , Fiske, S. T. , Koch, A. , & Yzerbyt, V. (2021). Navigating the social world: Toward an integrated framework for evaluating self, individuals, and groups. Psychological Review, 128(2), 290–314. 10.1037/rev0000262 32940512

[bjso70003-bib-0002] Adler, N. E. , Epel, E. S. , Castellazzo, G. , & Ickovics, J. R. (2000). Relationship of subjective and objective social status with psychological and physiological functioning: Preliminary data in healthy white women. Health Psychology, 19(6), 586–592. 10.1037//0278-6133.19.6.586 11129362

[bjso70003-bib-0003] Alcañiz‐Colomer, J. , Moya, M. , & Valor‐Segura, I. (2023). Not all poor are equal: the perpetuation of poverty through blaming those who have been poor all their lives. Curr Psychol, 42, 26928–26944. 10.1007/s12144-022-03804-6 PMC9533286 36213572

[bjso70003-bib-0004] Antonoplis, S. (2023). Studying socioeconomic status: Conceptual problems and an alternative path forward. Perspectives on Psychological Science, 18(2), 275–292. 10.1177/17456916221093615 35981108 PMC10018062

[bjso70003-bib-0005] Becker, J. C. , Kraus, M. W. , & Rheinschmidt‐Same, M. (2017). Cultural expressions of social class and their implications for group‐related beliefs and behaviors. Journal of Social Issues, 73(1), 158–174. 10.1111/josi.12209

[bjso70003-bib-0006] Bourdieu, P. (1979/1984). Distinction. A social critique of the judgment of taste. Harvard University Press.

[bjso70003-bib-0007] Bradley, H. (2014). Class descriptors or class relations? Thoughts towards a critique of savage et al. Sociology, 48(3), 429–436. 10.1177/0038038514520855

[bjso70003-bib-0008] Braun, V. , & Clarke, V. (2022). Conceptual and design thinking for thematic analysis. Qualitative Psychology, 9(1), 3–26. 10.1037/qup0000196

[bjso70003-bib-0009] Carballo‐Cruz, F. (2011). Causes and consequences of the Spanish economic crisis: Why the recovery is taking so long? Panoeconomicus, 58(3), 309–328. 10.2298/PAN1103309C

[bjso70003-bib-0010] Chambon, M. , Dalege, J. , Elberse, J. E. , & van Harreveld, F. (2022). A psychological network approach to attitudes and preventive behaviors during pandemics: A COVID‐19 study in the United Kingdom and the Netherlands. Social Psychological and Personality Science, 13(1), 233–245. 10.1177/19485506211002420 38603079 PMC8042407

[bjso70003-bib-0011] Champely, S. , Ekstrom, C. , Dalgaard, P. , Gill, J. , Weibelzahl, S. , Anandkumar, A. , Ford, C. , Volcic, R. , & De Rosario, H. (2017). pwr: Basic functions for power analysis . Software. https://cran.r‐project.org/web/packages/pwr/

[bjso70003-bib-0012] Connor, P. , Varney, J. , Keltner, D. , & Chen, S. (2021). Social class competence stereotypes are amplified by socially signaled economic inequality. Personality and Social Psychology Bulletin, 47(1), 89–105. 10.1177/0146167220916640 32441220

[bjso70003-bib-0013] Cuddy, A. J. C. , Fiske, S. T. , & Glick, P. (2008). Warmth and competence as universal dimensions of social perception: The stereotype content model and the BIAS map. Advances in Experimental Social Psychology, 40, 61–149. 10.1016/S0065-2601(07)00002-0

[bjso70003-bib-0014] Cuddy, A. J. C. , Fiske, S. T. , Kwan, V. S. Y. , Glick, P. , Demoulin, S. , Leyens, J.‐P. , Bond, M. H. , Croizet, J.‐C. , Ellemers, N. , Sleebos, E. , Htun, T. T. , Kim, H.‐J. , Maio, G. , Perry, J. , Petkova, K. , Todorov, V. , Rodríguez‐Bailón, R. , Morales, E. , Moya, M. , … Ziegler, R. (2009). Stereotype content model across cultures: Towards universal similarities and some differences. British Journal of Social Psychology, 48(1), 1–33. 10.1348/014466608X314935 19178758 PMC3912751

[bjso70003-bib-0016] Dalege, J. , Borsboom, D. , van Harreveld, F. , & van der Maas, H. L. J. (2017). Network analysis on attitudes: A brief tutorial. Social Psychological and Personality Science, 8(5), 528–537. 10.1177/1948550617709827 28919944 PMC5582642

[bjso70003-bib-0015] Dalege, J. , Borsboom, D. , van Harreveld, F. , van den Berg, H. , Conner, M. , & van der Maas, H. L. J. (2016). Toward a formalized account of attitudes: The causal attitude network (CAN) model. Psychological Review, 123(1), 2–22. 10.1037/a0039802 26479706

[bjso70003-bib-0017] Dalege, J. , Borsboom, D. , van Harreveld, F. , Waldorp, L. J. , & van der Maas, H. L. J. (2017). Network structure explains the impact of attitudes on voting decisions. Scientific Reports, 7(1), 4909. 10.1038/s41598-017-05048-y 28687776 PMC5501836

[bjso70003-bib-0018] Davidai, S. , & Wienk, M. N. (2021). The psychology of lay beliefs about economic mobility. Social and Personality Psychology Compass, 15(8), e12625. 10.1111/spc3.12625

[bjso70003-bib-0019] de Raadt, A. , Warrens, M. J. , Bosker, R. J. , & Kiers, H. A. L. (2021). A comparison of reliability coefficients for ordinal rating scales. Journal of Classification, 38, 519–543. 10.1007/s00357-021-09386-5

[bjso70003-bib-0020] DerSimonian, R. , & Laird, N. (1986). Meta‐analysis in clinical trials. Controlled Clinical Trials, 7(3), 177–188. 10.1016/0197-2456(86)90046-2 3802833

[bjso70003-bib-0021] Destin, M. , Rheinschmidt‐Same, M. , & Richeson, J. A. (2017). Status‐based identity: A conceptual approach integrating the social psychological study of socioeconomic status and identity. Perspectives on Psychological Science, 12(2), 270–289. 10.1177/1745691616664424 28346114

[bjso70003-bib-0022] Durante, F. , Fiske, S. T. , Kervyn, N. , Cuddy, A. J. C. , Akande, A. , Adetoun, B. E. , Adewuyi, M. F. , Tserere, M. M. , Ramiah, A. A. , Mastor, K. A. , Barlow, F. K. , Bonn, G. , Tafarodi, R. W. , Bosak, J. , Cairns, E. , Doherty, C. , Capozza, D. , Chandran, A. , Chryssochoou, X. , … Storari, C. C. (2013). Nations' income inequality predicts ambivalence in stereotype content: How societies mind the gap. British Journal of Social Psychology, 52(4), 726–746. 10.1111/bjso.12005 23039178 PMC3855559

[bjso70003-bib-0023] Durante, F. , Tablante, C. B. , & Fiske, S. T. (2017). Poor but warm, rich but cold (and competent): Social classes in the stereotype content model. Journal of Social Issues, 73(1), 138–157. 10.1111/josi.12208

[bjso70003-bib-0024] Easterbrook, M. J. , Kuppens, T. , & Grigoryan, L. (2023). Introduction to the special issue: Nuances of social class and socioeconomic status (Introducción a este monográfico: los matices del concepto de clase social y del nivel socioeconómico). International Journal of Social Psychology, 38(3), 493–507. 10.1080/02134748.2023.2239577

[bjso70003-bib-0025] Easterbrook, M. J. , Kuppens, T. , & Manstead, A. S. R. (2020). Socioeconomic status and the structure of the self‐concept. British Journal of Social Psychology, 59(1), 66–86. 10.1111/bjso.12334 31175690

[bjso70003-bib-0026] Elenbaas, L. , McGuire, L. , Ackerman, A. , Kneeskern, E. , Kinnard, L. , Farooq, A. , Law, F. , Makanju, D. , Ebert, K. , & Mistry, R. S. (2024). Social class group identity, intergroup attitudes, and views on social mobility and inequality in the UK and the US. Analyses of Social Issues and Public Policy. 10.1111/asap.12431 PMC11804895 39925984

[bjso70003-bib-0027] Evans, G. , & Mellon, J. (2016). Social class: Identity, awareness and political attitudes: why are we still working class? British Social Attitudes, 33. http://www.bsa.natcen.ac.uk/latest‐report/british‐social‐attitudes‐33/social‐class.aspx

[bjso70003-bib-0028] Evans, M. D. , & Kelley, J. (2004). Subjective social location: Data from 21 nations. International Journal of Public Opinion Research, 16(1), 3–38. 10.1093/ijpor/16.1.3

[bjso70003-bib-0031] Fiske, S. T. , & Markus, H. R. (2012). Facing social class: How societal rank influences interaction. Russell Sage Foundation.

[bjso70003-bib-0029] Fiske, S. T. , Cuddy, A. J. C. , & Glick, P. (2007). Universal dimensions of social cognition: Warmth and competence. Trends in Cognitive Sciences, 11(2), 77–83. 10.1016/j.tics.2006.11.005 17188552

[bjso70003-bib-0030] Fiske, S. T. , Cuddy, A. J. C. , Glick, P. , & Xu, J. (2002). A model of (often mixed) stereotype content: Competence and warmth respectively follow from perceived status and competition. Journal of Personality and Social Psychology, 82(6), 878–902. 10.1037/0022-3514.82.6.878 12051578

[bjso70003-bib-0032] García‐Castro, J. D. , García‐Sánchez, E. , Montoya‐Lozano, M. , & Rodríguez‐Bailón, R. (2022). The perception of economic inequality in everyday life: My friends with the most and least money. Asian Journal of Social Psychology, 25(1), 20–34. 10.1111/ajsp.12476

[bjso70003-bib-0033] García‐Sánchez, E. , García‐Castro, J. D. , Willis, G. B. , & Rodríguez‐Bailón, R. (2022). Percepción de desigualdad económica en la vida cotidiana e ideología política: Un estudio con jóvenes de España. Revista de Estudios Sociales, 79, 79. 10.7440/res79.2022.01

[bjso70003-bib-0034] García‐Sánchez, E. , Willis, G. B. , Rodríguez‐Bailón, R. , García‐Castro, J. D. , Palacio‐Sañudo, J. , Polo, J. , & Rentería‐Pérez, E. (2018). Perceptions of economic inequality in Colombian daily life: More than unequal distribution of economic resources. Frontiers in Psychology, 9, 1660. 10.3389/fpsyg.2018.01660 30237779 PMC6135891

[bjso70003-bib-0035] Grigoryan, L. , Bai, X. , Durante, F. , Fiske, S. T. , Fabrykant, M. , Hakobjanyan, A. , Javakhishvili, N. , Kadirov, K. , Kotova, M. , Makashvili, A. , Maloku, E. , Morozova‐Larina, O. , Mullabaeva, N. , Samekin, A. , Verbilovich, V. , & Yahiiaiev, I. (2020). Stereotypes as historical accidents: Images of social class in postcommunist versus capitalist societies. Personality and Social Psychology Bulletin, 46(6), 927–943. 10.1177/0146167219881434 31610737

[bjso70003-bib-0036] Hong, Y. Y. , Levy, S. R. , & Chiu, C. Y. (2003). The contribution of the lay theories approach to the study of groups. In S. R. Levy & C. Y. Chiu (Eds.), Lay theories and their role in the perception of social groups (pp. 98–106). Psychology Press.

[bjso70003-bib-0037] Horwitz, S. R. , & Dovidio, J. F. (2017). The rich—Love them or hate them? Divergent implicit and explicit attitudes toward the wealthy. Group Processes & Intergroup Relations, 20(1), 3–31. 10.1177/1368430215596075

[bjso70003-bib-0038] Jetten, J. , Peters, K. , Álvarez, B. , Casara, B. G. S. , Dare, M. , Kirkland, K. , Sánchez‐Rodríguez, Á. , Selvanathan, H. P. , Sprong, S. , Tanjitpiyanond, P. , Wang, Z. , & Mols, F. (2021). Consequences of economic inequality for the social and political vitality of society: A social identity analysis. Political Psychology, 42(S1), 241–266. 10.1111/pops.12800

[bjso70003-bib-0039] Jetten, J. , Wang, Z. , Steffens, N. K. , Mols, F. , Peters, K. , & Verkuyten, M. (2017). A social identity analysis of responses to economic inequality. Current Opinion in Psychology, 18, 1–5. 10.1016/j.copsyc.2017.05.011 29221504

[bjso70003-bib-0040] Jordan, J. A. , Lawler, J. R. , & Bosson, J. K. (2021). Ambivalent classism: The importance of assessing hostile and benevolent ideologies about poor people. Basic and Applied Social Psychology, 43(1), 46–67. 10.1080/01973533.2020.1828084

[bjso70003-bib-0041] Kay, A. C. , & Friesen, J. (2011). On social stability and social change: Understanding when system justification does and does not occur. Current Directions in Psychological Science, 20(6), 360–364. 10.1177/0963721411422059

[bjso70003-bib-0042] Kay, A. C. , & Jost, J. T. (2003). Complementary justice: Effects of “poor but happy” and “poor but honest” stereotype exemplars on system justification and implicit activation of the justice motive. Journal of Personality and Social Psychology, 85(5), 823–837. 10.1037/0022-3514.85.5.823 14599247

[bjso70003-bib-0043] Kay, A. C. , Jost, J. T. , Mandisodza, A. N. , Sherman, S. J. , Petrocelli, J. V. , & Johnson, A. L. (2007). Panglossian ideology in the service of system justification: How complementary stereotypes help us to rationalize inequality. Advances in Experimental Social Psychology, 39, 305–358. 10.1016/S0065-2601(06)39006-5

[bjso70003-bib-0044] Kervyn, N. , Yzerbyt, V. , & Judd, C. M. (2010). Compensation between warmth and competence: Antecedents and consequences of a negative relation between the two fundamental dimensions of social perception. European Review of Social Psychology, 21(1), 155–187. 10.1080/13546805.2010.517997

[bjso70003-bib-0045] Klandermans, B. (2014). Identity politics and politicized identities: Identity processes and the dynamics of protest. Political Psychology, 35(1), 1–22.

[bjso70003-bib-0047] Kraus, M. W. , & Stephens, N. M. (2012). A road map for an emerging psychology of social class. Social and Personality Psychology Compass, 6(9), 642–656. 10.1111/j.1751-9004.2012.00453.x

[bjso70003-bib-0046] Kraus, M. W. , Park, J. W. , & Tan, J. J. X. (2017). Signs of social class: The experience of economic inequality in everyday life. Perspectives on Psychological Science, 12(3), 422–435. 10.1177/1745691616673192 28544871 PMC5453398

[bjso70003-bib-0074] Kraus, M. W. , Piff, P. K. , Mendoza‐Denton, R. , Rheinschmidt, M. L. , & Keltner, D. (2012). Social class, solipsism, and contextualism: How the rich are different from the poor. Psychological Review, 119(3), 546. 10.1037/a0028756 22775498

[bjso70003-bib-0048] Krippendorff, K. (2018). Content analysis: An introduction to its methodology. Sage Publications.

[bjso70003-bib-0049] Lickel, B. , Hamilton, D. L. , & Sherman, S. J. (2003). Elements of a lay theory of groups: Types of groups, relational styles, and the perception of group entitativity. In Y. Y. Hong , S. R. Levy , & C. Y. Chiu (Eds.), Lay theories and their role in the perception of social groups (pp. 129–140). Psychology Press.

[bjso70003-bib-0050] Malo, M. Á. (2015). Labour market measures in Spain 2008–13: The crisis and beyond (Research Department Working Paper No. 10). International Labour Office. https://www.ilo.org/wcmsp5/groups/public/‐‐‐dgreports/‐‐‐inst/documents/publication/wcms_449933.pdf

[bjso70003-bib-0051] Manstead, A. S. R. (2018). The psychology of social class: How socioeconomic status impacts thought, feelings, and behaviour. British Journal of Social Psychology, 57(2), 267–291. 10.1111/bjso.12251 29492984 PMC5901394

[bjso70003-bib-0052] Marsden, P. V. , Smith, T. W. , & Hout, M. (2020). Tracking US social change over a half‐century: The general social survey at fifty. Annual Review of Sociology, 46(1), 109–134. 10.1146/annurev-soc-121919-054838

[bjso70003-bib-0053] Moreno‐Bella, E. , Willis, G. B. , & Moya, M. (2019). Economic inequality and masculinity–femininity: The prevailing perceived traits in higher unequal contexts are masculine. Frontiers in Psychology, 10, 1590. 10.3389/fpsyg.2019.01590 31428004 PMC6688552

[bjso70003-bib-0054] Peters, K. , & Jetten, J. (2023). How living in economically unequal societies shapes our minds and our social lives. British Journal of Psychology, 114(2), 515–531. 10.1111/bjop.12632 36708128

[bjso70003-bib-0055] Pew Research Center . (2012). Yes, the rich are different . http://www.pewsocialtrends.org/files/2012/08/sdt‐rich‐poor‐082712.pdf

[bjso70003-bib-0056] R Core Team . (2017). R: A language and environment for statistical computing. R Foundation for Statistical Computing. https://www.R‐project.org/

[bjso70003-bib-0057] Rinn, R. , Ludwig, J. , Fassler, P. , & Deutsch, R. (2023). Cues of wealth and the subjective perception of rich people. Current Psychology, 42(31), 27442–27457. 10.1007/s12144-022-03763-y

[bjso70003-bib-0058] Royo, S. (2020). The causes and legacy of the great recession in Spain. In D. Muro & I. Lago (Eds.), The Oxford handbook of Spanish politics. Oxford University Press. 10.1093/oxfordhb/9780198826934.013.8

[bjso70003-bib-0059] Sainz, M. , Lobato, R. M. , & Jiménez‐Moya, G. (2021). Spanish adaptation of the ambivalent classism inventory (ACI). Revista Latinoamericana de Psicología, 53, 164–171. 10.14349/rlp.2021.v53.18

[bjso70003-bib-0060] Sánchez‐Rodríguez, Á. , Moreno‐Bella, E. , & García‐Sánchez, E. (2023). Mapping gender stereotypes: A network analysis approach. Frontiers in Psychology, 14, 1193866. 10.3389/fpsyg.2023.1193866 37533725 PMC10393260

[bjso70003-bib-0061] Sayans‐Jiménez, P. , Harreveld, F. , Dalege, J. , & Rojas Tejada, A. J. (2019). Investigating stereotype structure with empirical network models. European Journal of Social Psychology, 49(3), 604–621. 10.1002/ejsp.2505

[bjso70003-bib-0062] Shorrocks, A. , Davies, J. , & Lluberas, R. (2020). Global wealth report . https://www.credit‐suisse.com/media/assets/corporate/docs/about‐us/research/publications/global‐wealth‐report‐2020‐en.pdf

[bjso70003-bib-0063] Spears, R. (2011). Group identities: The social identity perspective. In S. J. Schwartz , K. Luyckx , & V. L. Vignoles (Eds.), Handbook of identity theory and research (pp. 201–224). Springer. 10.1007/978-1-4419-7988-9_9

[bjso70003-bib-0064] Sussman, L. , Dubofsky, D. , Levitan, A. S. , & Swidan, H. (2014). Good rich, bad rich: Perceptions about the extremely wealthy and their sources of wealth. International Journal of Business and Social Research, 4(8), 44–58.

[bjso70003-bib-0065] Tanjitpiyanond, P. , Jetten, J. , & Peters, K. (2022). How economic inequality shapes social class stereotyping. Journal of Experimental Social Psychology, 98, 104248. 10.1016/j.jesp.2021.104248

[bjso70003-bib-0066] Tanjitpiyanond, P. , Jetten, J. , Peters, K. , Ashokkumar, A. , Barry, O. , Billet, M. , Becker, M. , Booth, R. W. , Castro, D. , Chinchilla, J. , Costantini, G. , Dejonckheere, E. , Dimdins, G. , Erbas, Y. , Espinosa, A. , Finchilescu, G. , Gómez, Á. , González, R. , Goto, N. , … Yeung, V. W. L. (2023). A 32‐society investigation of the influence of perceived economic inequality on social class stereotyping. European Journal of Social Psychology, 53(2), 367–382. 10.1002/ejsp.2908

[bjso70003-bib-0067] Tao, S. , Ha, L. , & Yuan, C. (2016). The complementary stereotypes about the rich and the poor: A study in China. Open Journal of Social Sciences, 4(11), 113–122. 10.4236/jss.2016.411009

[bjso70003-bib-0068] Turner, J. C. , Hogg, M. A. , Oakes, P. J. , Reicher, S. D. , & Wetherell, M. S. (1987). Rediscovering the social group: A self‐categorization theory. Blackwell.

[bjso70003-bib-0070] Viechtbauer, W. (2010). Conducting meta‐analyses in R with the metafor package. Journal of Statistical Software, 36, 1–48. 10.18637/jss.v036.i03

[bjso70003-bib-0071] Willis, G. B. , García‐Sánchez, E. , Sánchez‐Rodríguez, Á. , García‐Castro, J. D. , & Rodríguez‐Bailón, R. (2022). The psychosocial effects of economic inequality depend on its perception. Nature Reviews Psychology, 1(5), 301–309. 10.1038/s44159-022-00044-0

[bjso70003-bib-0072] World Economic Forum . (2019). Poverty . https://www.worldbank.org/en/topic/poverty

[bjso70003-bib-0073] Wright, E. O. (2015). Understanding class. Verso.

