# Supplementary material for: Lay representations of social class: A mixed methods approach to wealth‐based group perceptions and stereotypes
Source: Br J Soc Psychol. 2025 Jun 27;64(3):e70003. doi: 10.1111/bjso.70003 (PMC12205169; doi:10.1111/bjso.70003)
Supplement: Supplementary file 1 — Appendix S1 [file BJSO-64-0-s001.docx]

**Supplementary Materials**

**pertaining to**

**Social class representations: Analysing wealth-based groups stereotypes through a mixed-method approach**

This section contains supplementary material in addition to the main manuscript.

Study 1: Additional measures (Figure S1), list of traits, list of traits after review by expert judges, full procedure, preliminary results, coding framework (Table S1, S2), results (Table S3), pictograms (Figure S2).

Study 2: Procedural material (Figures S3, S4, S5, S6, S7), and tables summarising results from Study 2 (Table S4, S5, S6, S7).

Studies 3a and 3b: Procedure material (Figures S8, S9, S10, S11, S12), and tables summarising results from Study 3b (Figures S13; Tables S8, S9, S10 y S11)

All materials are available at the following OSF link: <https://osf.io/549mq/?view_only=68bbb37826064539813a6f5f4f371c6d>

**Study 1**

**Additional measures**

After the end of the interview the participants answered some exploratory measures

“Remember the economic reality that exists in the fictional society "Cratania". This distribution of wealth, that is, how wealth is distributed between the poorest people and the richest people, is the distribution of wealth that currently exists in the world. The graph that represents how people distribute wealth is real, it belongs to the distribution of wealth in the world. Remember that the graph represents all the inhabitants of the world. That is, the 100 people that are represented in the graph are equivalent to the world population. Think about your economic situation if you are economically independent, or that of your family if you are not, and imagine that one of the people represented in the graph is you. Think about the distribution of wealth that we present to you and imagine where you would be in that distribution:”

**Group identification (99%, middle class, upper class and working class).** First, participants read the definition of these identities. In the case of 99% identity: “The term 99% represents the majority of the world’s population (the 99%) against a very small percentage (1%) that owns half of the planet’s wealth (if wealth were a pie cut in two, the 1% richest gets one half while the other corresponds to 99% of the world’s inhabitants)”, working class identity : “The term working class designates the group of workers who work in exchange for a salary in opposition to the ruling class that owns the majority of the property of economic resources”, middle class: “The term middle class designates the group of people who belong to the social class that is applied to people with an average socioeconomic level which is between the lower class and the upper class”, upper class “The term upper class designates the group of people who belong to the social class that is applied to people with a high socioeconomic level which is above the rest of the social strata”.

After reading the descriptions participants indicated the extent to which they identified with 99%, working-class middle class, and upper class:

- I identify with...
- I feel connected to...
- I feel in solidarity with...
- I feel committed to...
- I often think about the fact that I am part of...
- I t is an important part of my identity to be part of...
- It's a big part of how I see myself being a part of...

**Intolerance toward economic inequality.** Being 1 “Strongly disagree” and 7 “Strongly agree”, to what extent would you rate the following statements?

- The negative consequences of economic inequality have been greatly exaggerated.
- Economic inequality is causing many of the problems in Spain.
- I am very concerned about the degree of economic inequality that exists in Spain.
- Economic inequality is not a problem.
- We must do everything possible to reduce the economic inequality that exists in Spain today.

**Collective action intentions.** Next, we ask you to answer with what probability you would participate in actions to reduce economic inequality. Being 1 "Never" and 7 "Very often", to what extent would you be willing to carry out the following actions?

- I would vote for political parties whose priorities are reducing economic inequality.
- I would contact political representatives to promote policies to combat economic inequality.
- I would donate money to a political party or organization that wants to combat economic inequality.
- I would belong to a political party, union, or organization against economic inequality.
- I would participate in union or political group activities to combat economic inequality.
- I would participate in peaceful demonstrations demanding the reduction of economic inequality.
- I would engage in nonviolent civil disobedience to protest laws that favour economic inequality.
- I would distribute political material against economic inequality.
- I would boycott products that maintain economic inequality.
- I would sign petitions against economic inequality.
- He would be active in movements against economic inequality.

**Sociodemographic measures.** Finally, some sociodemographic data were requested: subjective socioeconomic status with the Scale of Subjective Social Status (SES, Adler et al., 2000), objective economic status, political orientation, educational level, gender and age.

**Procedure**

**Graph counterbalancing.** To control for possible aesthetic influences on participants’ interpretation of the graphs, a counterbalancing procedure was included. Specifically, participants were randomly assigned to view one of the two graph formats—a bar chart or an exponential curve—both depicted the same wealth distribution patterns (Graph A = 47 participants, Graph B = 43 participants; see Figure 1). This experimental control ensured that any differences in categorisation were due to conceptual distinctions and not to the graphical presentation. Additionally, these graphs served as a visual aid during the interview.

**Figure 1**

*Graphs that were presented to the participants to represent the distribution of wealth in two different ways.*

Graph A (bars) Graph B (exponential curve)


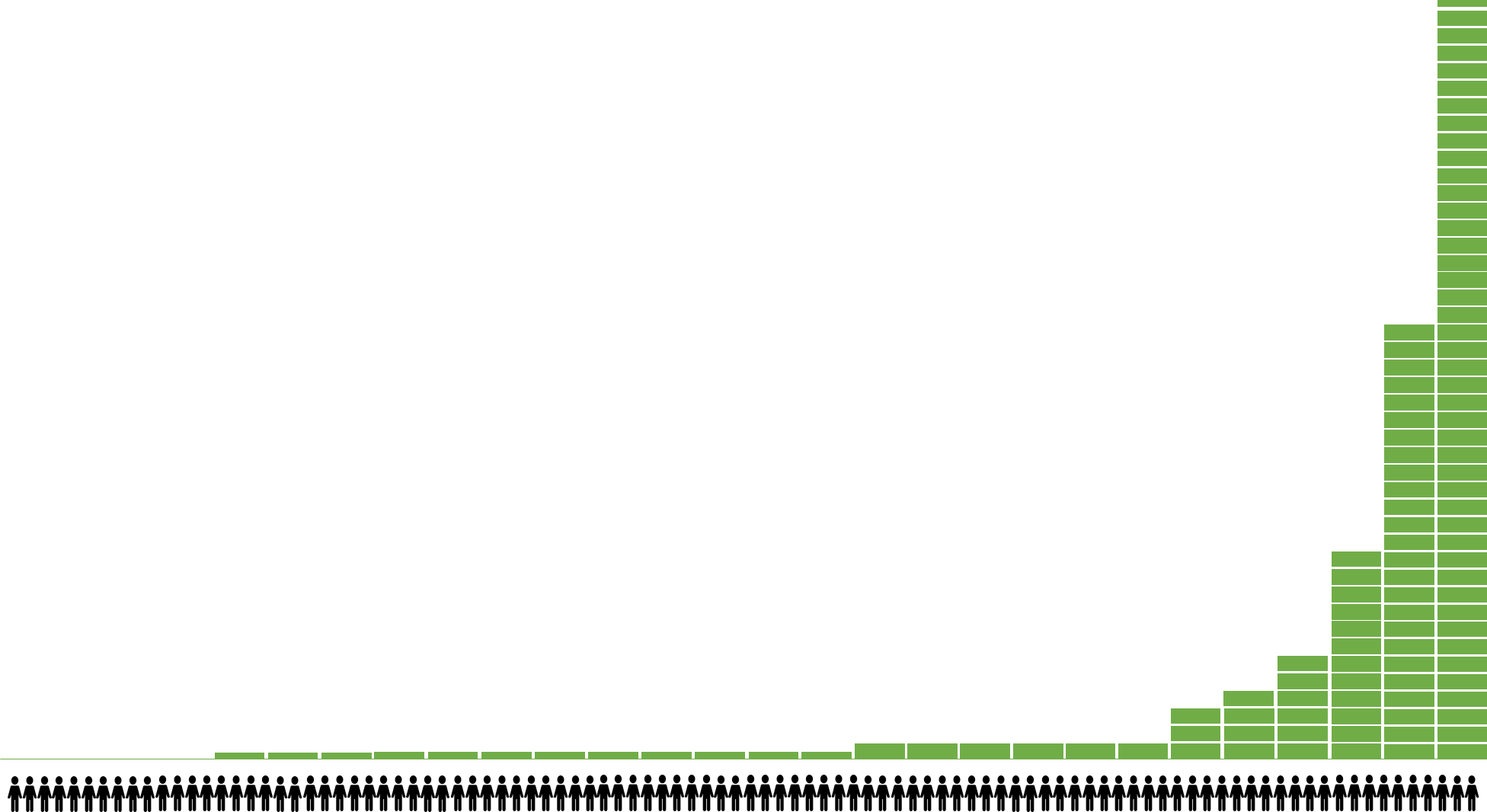

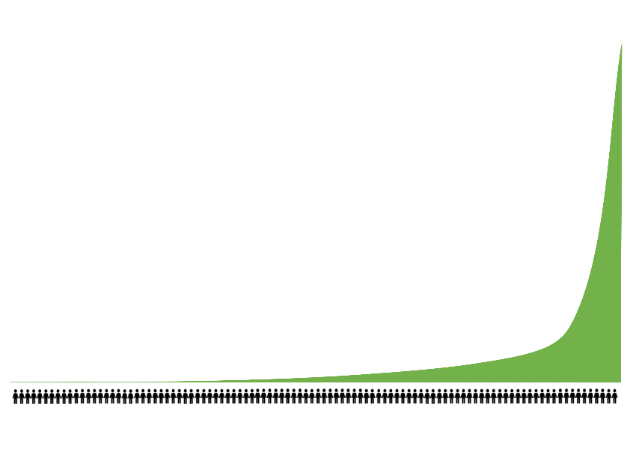


After viewing the assigned graph, participants rated perceived inequality using a Likert scale from 1 (Not unequal at all/ Not egalitarian) to 7 (Very unequal/Very egalitarian). They responded to two questions: (1) Overall, to what extent do you think the society presented has an unequal distribution of resources?” and (2) Overall, to what extent do you think the society presented has an egalitarian distribution of resources?” (the second item was reverse-coded). This measure was included to assess whether different graphical representations of the same wealth distribution elicited similar perceptions of inequality, ensuring that differences in categorisation were not due to disparities in perceived inequality across formats.

**Preliminary results**

We compared whether both economic inequality graphs (A = Bars graph; B = Curve graph; Figure S1) were perceived similarly and if this influenced the perception of inequality or the number of social classes created by the participants. For this reason, we conducted two independent sample t-tests. Results showed that the perception of economic inequality was the same in both graphs (*M* = 6.34, *SD* = .75; *M* = 6.18, *SD* = .96; *t*(88) = .854, *p* = .198).

Importantly, the number of groups formed was not associated with the key dependent variables: intolerance towards economic inequality (r = -.01, p = .904) and collective actions (r = -.08, p = .482). Nor is it associated with the qualitative content; terms used for the groups or the negative/positive traits of each group (negative traits: r = .03 p = .807; positive traits: r =.02 p = 820).

**Figure S1**

*Graphs that we showed to the participants to represent the distribution of wealth in two different ways.*


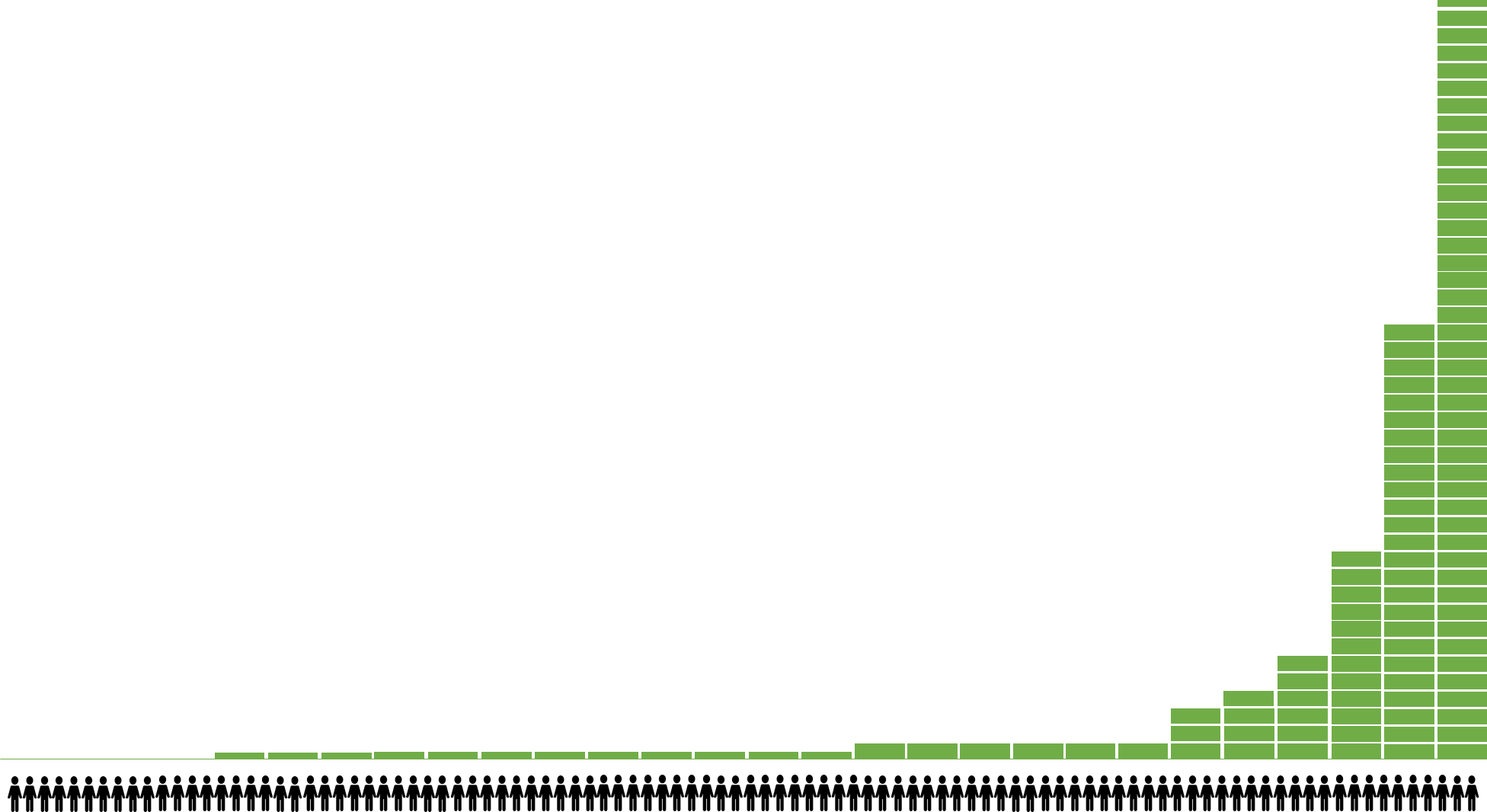

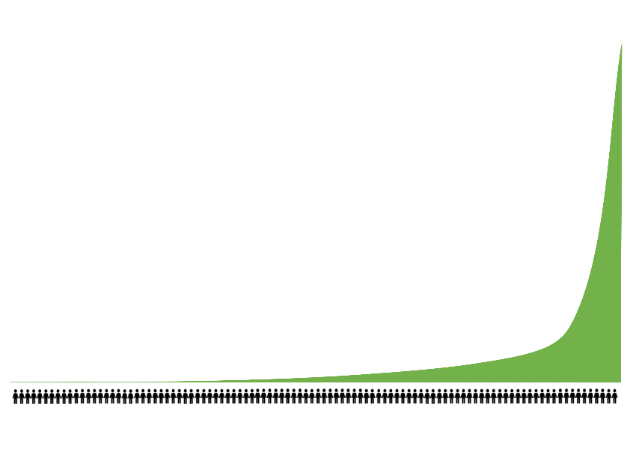


**Pictograms**

**Figure S2.**

*Some examples of participants' interactions with the graphs during the interview*

**
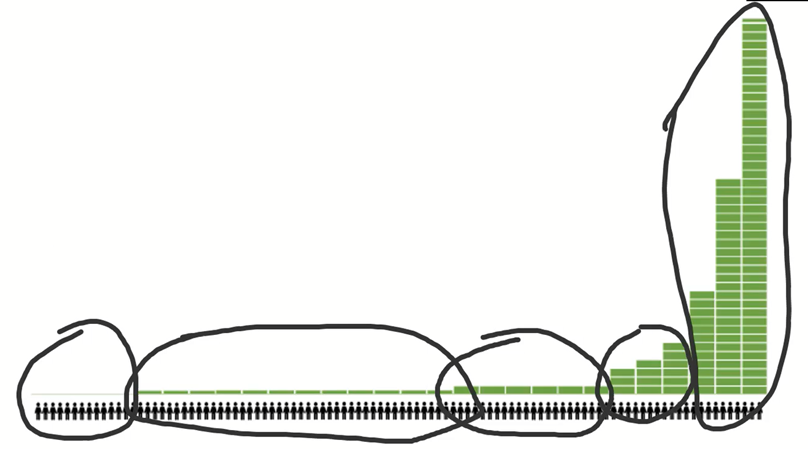
**

**
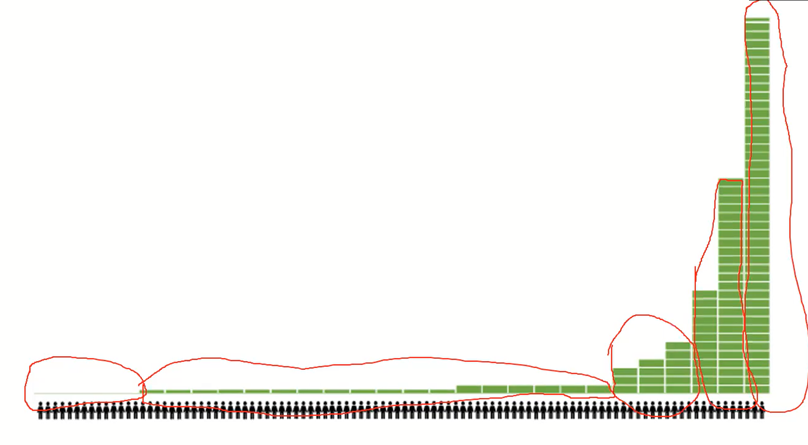
**

**
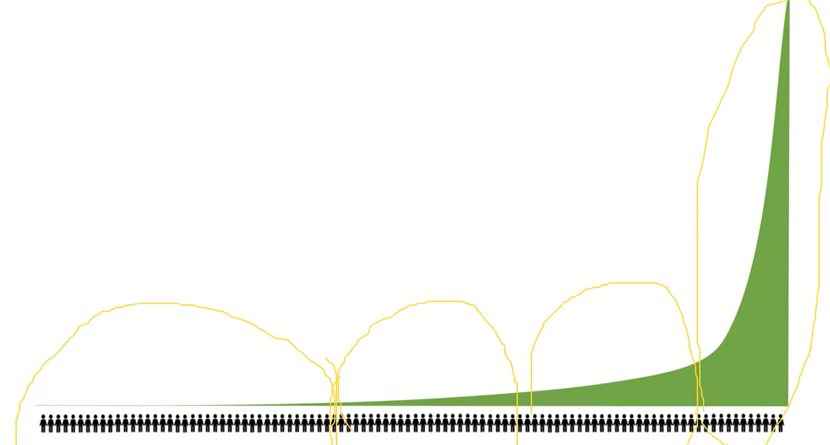
**

**
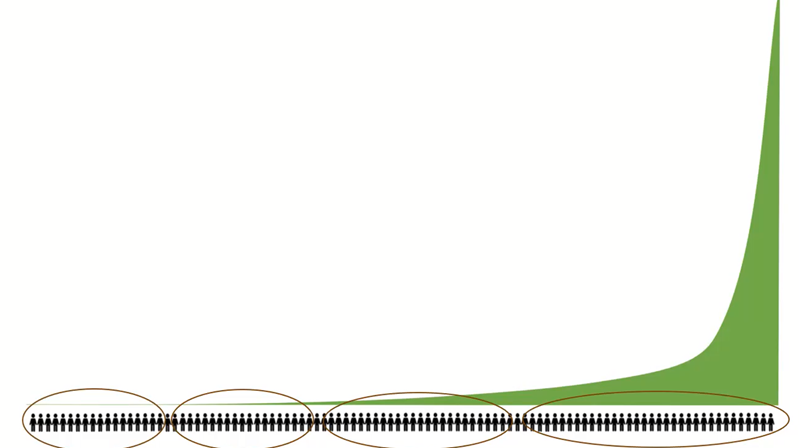
**

**
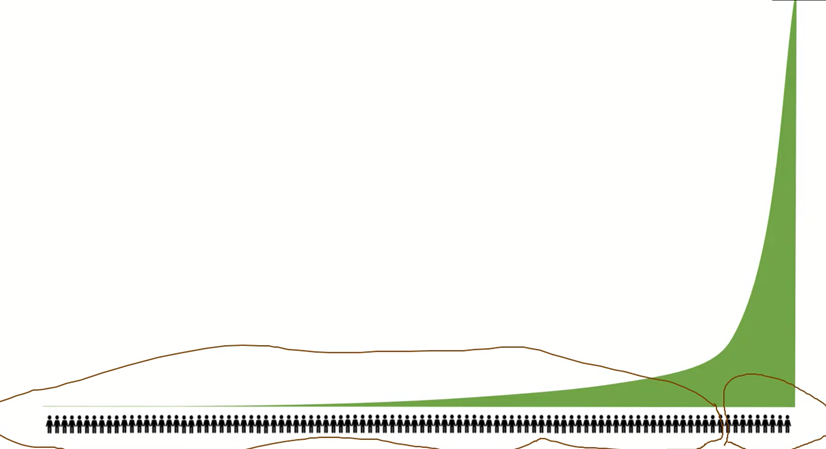
**

**
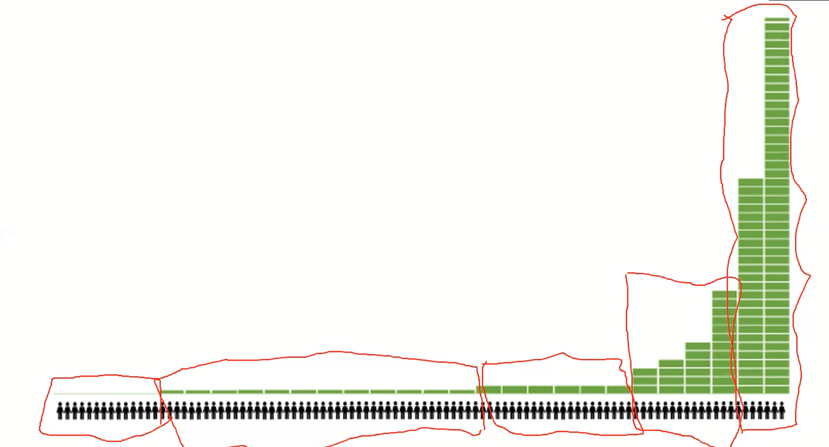
**

**
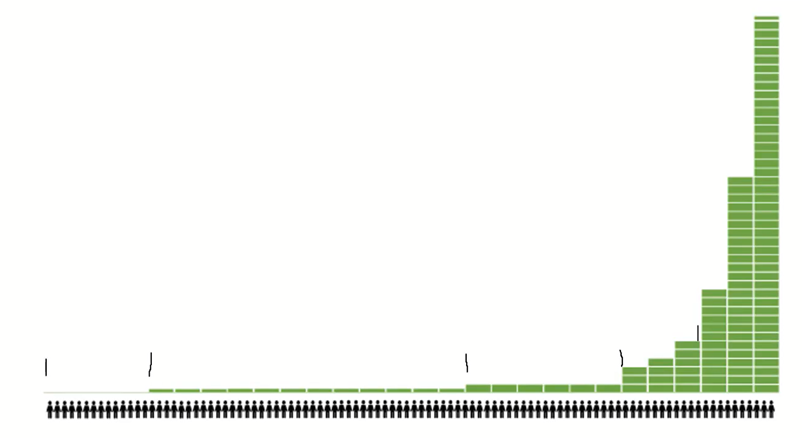
**

**
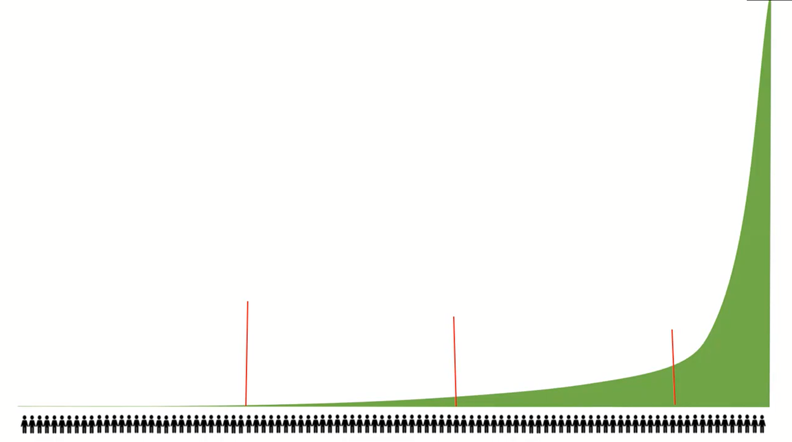
**

**
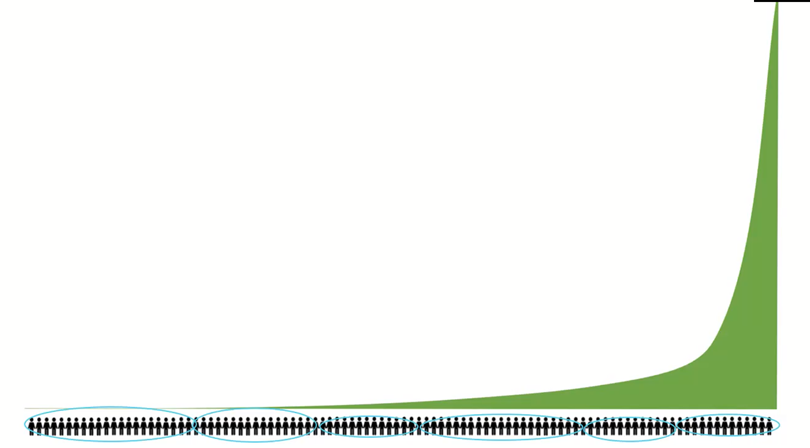
**

**Coding framework**

**Table S1**

*Categorical framework*

| **Category**  **(with definition)** | **Subcategory** | **Indicators and codes with examples** | **Frequency** |
| --- | --- | --- | --- |
| **Wealth-based groups**  Groups defined by a set of individuals have in common the wealth they possess. Additionally, the people comprising each group share resources and culture than make them like each other and distinguish them from other groups. This is also referred to as “SES” or “social class”. | - Poor - Low and working classes - Middle classes - Upper classes - Rich and Beyond | “The first one would say homeless, the second one would be poor, a bit poor, the third one would be average, normal, the fourth one would be wealthy, and the fifth one would be millionaires or rich” (P24)  “Very poor, poor, working class, upper middle class, rich or high class” (P46). | 1156 |
| **Traits**  The terms used to define personally, psychologically, and culturally the groups of people categorised based on wealth. These terms can be positive, negative, or neutral depending on the affective attitudes towards them. | - Positive - Negative - Neutral | “I believe that the non-wealthy are humble in the sense that, having nothing, they settle for very little. The normalised society, I would describe as ambitious, somewhere between humble and ambitious because some people may think, "Okay, what I have is enough, and I don't need much more," but there's another part of society that wants to increase their wealth, climb the social ladder, and always wants more. The wealthy would be very ambitious, and I think they would be less generous. What's mine is mine, and I want my wealth” (P25).  “The lower middle class and the poor are more honest because they have lived a different reality that the rich or the upper class have not experienced. So, I believe they have more camaraderie and empathy. Some of the upper-class individuals, not all, will be a bit selfish or discriminate against the poor. The middle-class group, I think, will have a bit of everything. Some will empathise more with the lower class because they see themselves reflected in them, while others will discriminate and try to pretend, they have more” (P59). | 447 |
| **Resources**  Resources that enable the lifestyle of wealth-based groups (e.g., properties, housing, clothing). Their resources can be quantified based on quantity or status. | - Nothing or absence - Basic or restricted - Moderate or normal - Many   &   - High status - Low status | “In the lower-class group, they probably have housing, likely social housing” (P38)  “The middle-class individuals do have a house, and maybe it’s not fully paid off, but they manage to make ends meet. They also have a vehicle. Moreover, if they have a job, it’s a stable one with a good salary, allowing them to afford their expenses. They can treat themselves to something nice every month, buy clothes, dine out a fancy restaurant every weekend, and go on vacation during the summer” (P50) | 793 |
| **Culture**  Set of knowledge, beliefs, values, norms, traditions, customs, artistic expressions, and symbolic manifestations that characterise a group of people and are influenced by the wealth that this group possess |  | “…and people with more money, well, they might listen to more refined things, like classical music and such…” (P5)  “People from the lower class, well, we could say that they have hobbies. I don’t know, maybe children like playing soccer because they can afford it, they can buy a ball, for example…” (P14)  “…there is more drug consumption, or consumption of trashy TV, or junk food…” (P64) | 108 |
| **Differences and similarities**  Perception of common goals or values and different experiences among groups |  | “There are two extremes. One has nothing, and the other one, I think, has everything” (P23)  “They are similar because they are people, but what sets them apparat is the way of life they have” (P11) | 56 |
| **Contact and relation**  Forms of intergroup contact between wealth-based groups, that is, the possibility of interactions between individuals belonging to different groups and the opportunities for establishing relationships. |  | “A rich person, with another rich person…I imagine that they only associate with people who have money…I think the rich always associate everything with money” (P1)  “…but the two extremes are the ones who have the least interaction, so there may be empathy, but not psychical interaction with them” (P64) | 30 |

Abbreviations: *(P)*., Participant number

**Table S2**

*Frequencies for the subcategories of the first main category: wealth-based group*

| **Categories** | **Subcategories** | **Labels^2^** | **Frequency** | **Percentage** |
| --- | --- | --- | --- | --- |
| **Wealth-based group** | Poor | Poor, poverty, homeless, extreme poverty, poorest, less poor, poor (just enough), intermediate poor, less privileged class, low economic resources, not so poor, disadvantaged, maximum poverty, super poor, needy, small poverty, scarce, not very poor. | 322 | 27.85% |
|  | Low-and Working-Classes | Lower class, working class, workers, working people, lumpen, precarious, very low, resigned, limited, non-existent wealth, low, insufficient wealth, very low economic level, fairly low economic level, underprivileged, labourers, not wealthy, very low class, limited wealth, unlucky, marginalised. | 129 | 11.16% |
|  | Middle-Classes | Middle class, middle, upper-middle class, normal, lower-middle, intermediate, lower-middle class, adequate, insufficient wealth, wealthy, lower bourgeois, conformist, stable, middle class, semi-privileged, moderate, normal economic level, normalised society, more wealth, middle economic level, normative, common, mediocre, in the middle, lucky, bourgeois, less wealth. | 303 | 26.21% |
|  | Upper-Classes | Upper class, high class, wealthy, privileged, excessive, powerful, dominant, most favoured class, billionaires, arrogant, politicians, high economic resources, affluent class, very privileged, wealthy, high wealth, low rich, engineers, soccer players. | 166 | 14.36% |
|  | Rich and Beyond | Rich, very rich, millionaires, very high class, royalty, high economic level, intermediate rich, rich class, high bourgeoisie, fortunate, very high economic level, excessive wealth, rich class, rotten in money, extreme wealth, more than rich, high rich, super-rich, very high wealth. | 236 | 20.42% |
| Total |  |  | 1156 | 100% |

**Results**

**Traits extracted from the qualitative study prior to the expert judgement (English translation):**

- Wasteful
- Educated
- Arrogant
- Emaciated physical appearance
- Classist
- Competent
- Desperate
- Extravagant
- Influential
- Unconcerned
- Proud
- Adapts to the environment
- Sophisticated
- Haughty
- Greedy
- Compares him/herself with those below
- Conformist
- Happy-go-lucky
- Non-conformist
- Evil
- Not status-conscious
- Financially concerned
- Concerned about one's inner circle
- No class consciousness
- Superciliousness
- Hardworking
- Calm
- Varied
- Kind
- Ambitious
- Basic
- Well-behaved
- Approachable
- Competitive
- Challenged
- Class-conscious
- Delinquent
- Unlucky
- Distrustful
- Different way of being
- Egocentric
- Selfish
- Empathetic
- Familiar
- Frivolous
- Hypocritical
- Homogeneous
- Honest
- Humble
- Indifferent
- Likes family/friends
- They have enough to spare
- Materialistic
- Fear of the poor
- Not caring
- Not sociable
- Noble
- Unambitious
- Relationships of convenience
- Religious
- Servant
- Sociable
- Supportive
- Survivor
- Susceptible
- Sadness
- Values things

**Main category 2: Traits selected after expert judgement, revised and adapted to English**

**Table S3**

*Frequencies and percentages of codes of the second main category: traits*

| **Main category: Traits** | **Subcategories** | **Codes*** | **Frequency** | **Per cent** |
| --- | --- | --- | --- | --- |
|  | Negatives (29) | Haughty | 40 | 15,75% |
|  |  | Selfish | 33 | 12,19% |
|  |  | Class-unconscious | 23 | 9,05% |
|  |  | Conformist | 20 | 7,87% |
|  |  | Materialistic | 18 | 7,09% |
|  |  | Arrogant | 15 | 5,91% |
|  |  | Greedy | 11 | 4,33% |
|  |  | Economically concerned | 10 | 3,94% |
|  |  | Pretentious | 10 | 3,94% |
|  |  | Sad | 8 | 3,15% |
|  |  | Careless | 8 | 3,15% |
|  |  | Unsociable | 7 | 2,76% |
|  |  | Bad | 6 | 2,36% |
|  |  | Indifferent | 6 | 2,36% |
|  |  | Opportunistic/self-interest | 5 | 1,97% |
|  |  | Desperate | 4 | 1,58% |
|  |  | Delinquent | 4 | 1,58% |
|  |  | Wasteful | 3 | 1,18% |
|  |  | Unambitious | 3 | 1,18% |
|  |  | Classist | 2 | 0,79% |
|  |  | Extravagant | 2 | 0,79% |
|  |  | Impressionable | 2 | 0,79% |
|  |  | Compares themselves to lower groups | 2 | 0,79% |
|  |  | Unlucky | 2 | 0,79% |
|  |  | Distrustful | 2 | 0,79% |
|  |  | Frivolous | 2 | 0,79% |
|  |  | Hypocrite | 2 | 0,79% |
|  |  | Fear of the poor | 2 | 0,79% |
|  |  | Susceptible | 2 | 0,79% |
| Total |  |  | 254 | 100 |
|  | Positives (20) | Humble | 192 | 27,08% |
|  |  | Kind | 71 | 10,06% |
|  |  | Happy | 64 | 9,09% |
|  |  | Value | 58 | 8,24% |
|  |  | Solidary | 56 | 7,92% |
|  |  | Empathic | 39 | 5,51% |
|  |  | Hardworking | 33 | 4,58% |
|  |  | Educated | 32 | 4,51% |
|  |  | Class-conscious | 30 | 4,29% |
|  |  | Distinct | 25 | 3,52% |
|  |  | Familiar | 22 | 3,15% |
|  |  | Competent | 21 | 2,91% |
|  |  | Approachable | 18 | 2,58% |
|  |  | Survivors | 9 | 1,26% |
|  |  | Sociable | 9 | 1,23% |
|  |  | Helpful | 8 | 1,16% |
|  |  | Competitive | 7 | 0,94% |
|  |  | Honest | 6 | 0,82% |
|  |  | Calm | 5 | 0,76% |
|  |  | Diverse | 3 | 0,40% |
| Total |  |  | 710 | 100% |

Note: *Economically concerned and educated were excluded from the main analyses because they were descriptions rather than central traits of the stereotype.

**Study 2**

***Procedural material***

Participants were informed that they would be shown five-word clouds containing words that people commonly use to refer to wealth-based groups or social classes. The terms used for each word cloud were the labels identified in Study 1 for each subcategory: (1) poor, (2) low and working classes, (3) middle classes, (4) upper classes, (5) rich and beyond. Participants were not shown these labels, only the content of the word clouds. However, to facilitate the process, participants created their own label for each word cloud. In this way, when they had to respond to the measures, the word cloud and the term they had developed themselves appeared.

**Figure S3**

*Word cloud representing the codes contained in the “poor” subcategory.*


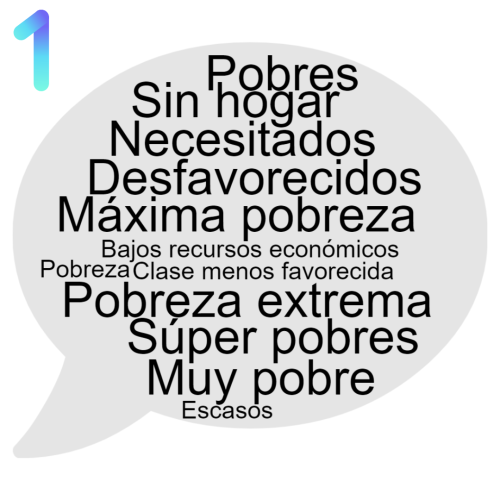


**Figure S4**

*Word cloud representing the codes contained in the “low and wornking classes” subcategory.*


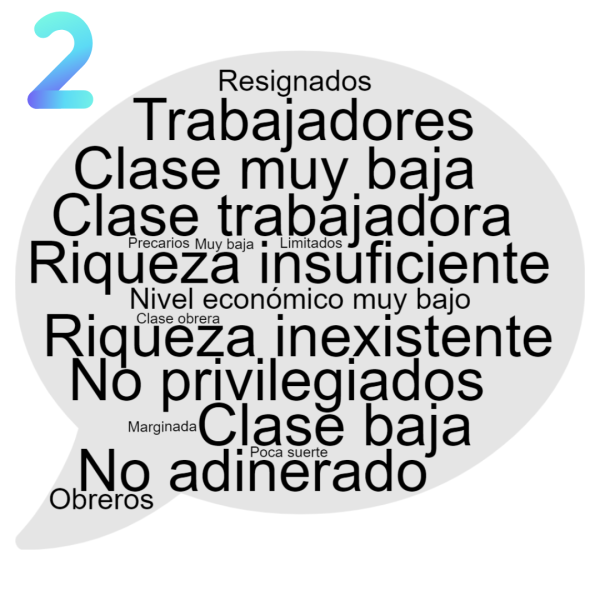


**Figure S5**

*Word cloud representing the codes contained in the “middle classes” subcategory.*


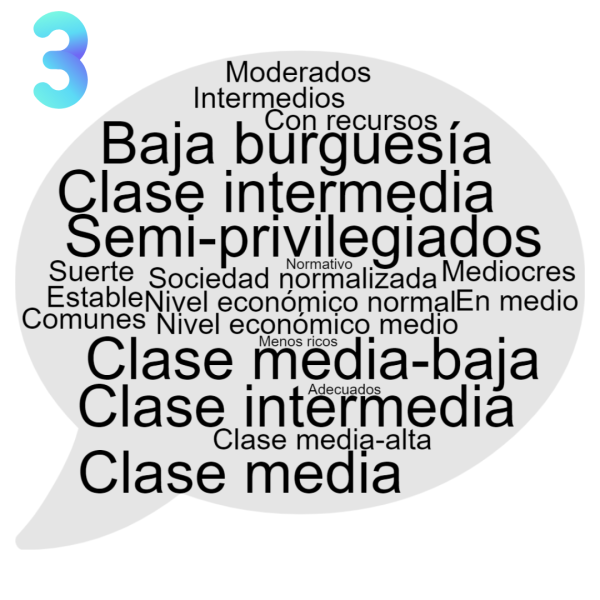


**Figure S6**

*Word cloud representing the codes contained in the “upper classes” subcategory.*


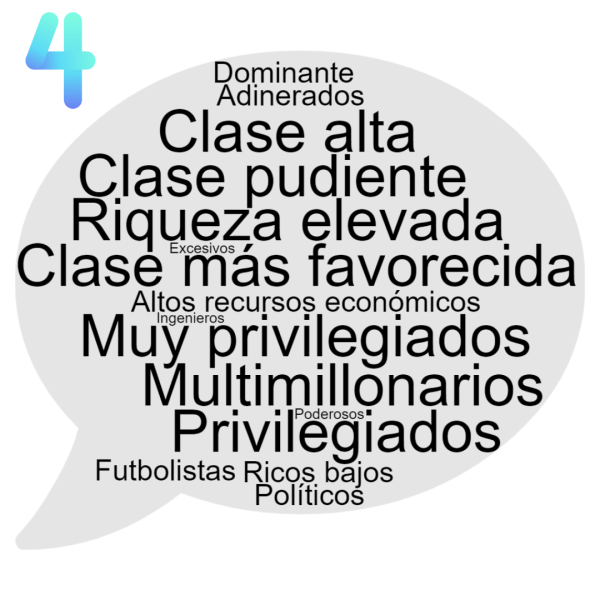


**Figure S7**

*Word cloud representing the codes contained in the “rich and beyond” subcategory.*


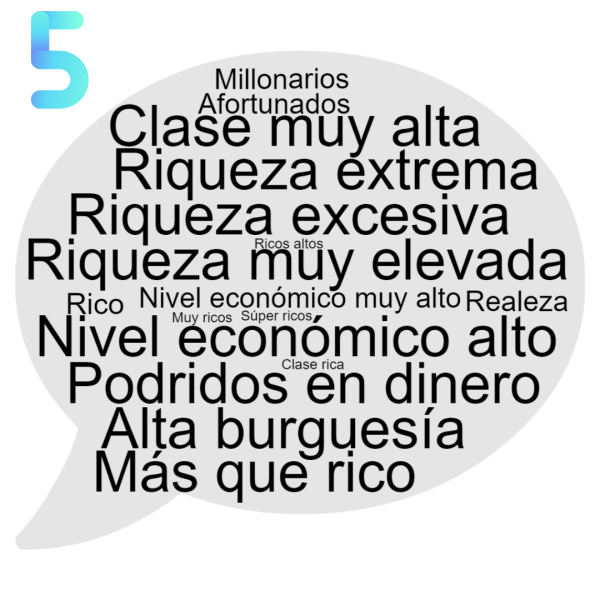


**Results**

**Perception of wealth-based groups in terms of negative valence traits.** The post hoc Bonferroni tests revealed that all the associations between negative traits and wealth-based groups were statistically significant, except for the negative trait “distrustful” (Table S4). After inspecting the pattern of the association, we found that most of the negative traits were linked to rich-and-beyond and upper-class compared to the remaining group (Table S5).

**Table S4**

*Chi-square test showing the associations between groups (poor, low and working classes, middle classes, upper classes, and rich an beyond) and negative traits*

| **Negative Trait*** | **χ2** | **gl** | **Cramer’s V** | **odds ratio (OR)** |
| --- | --- | --- | --- | --- |
| Haughty | 213.92** | 4 | .65 | 5.77 |
| Selfish | 230.68** | 4 | .68 | 11.35 |
| Class-unconscious | 63.46** | 4 | .36 | 0.34 |
| Conformist | 89.70** | 4 | .42 | 0.56 |
| Materialistic | 236.92** | 4 | .69 | 16.82 |
| Arrogant | 271.36** | 4 | .74 | -13.33 |
| Greedy | 206.88** | 4 | .64 | 4.69 |
| Economically concerned | 256.44** | 4 | .72 | -47.16 |
| Pretentious | 266.30** | 4 | .73 | -17.41 |
| Sad | 130.80** | 4 | .51 | 1.09 |
| Careless | 18.50** | 4 | .19 | .08 |
| Unsociable | 65.35** | 4 | .36 | .35 |
| Bad | 98.34** | 4 | .44 | .64 |
| Indifferent | 1.37** | 4 | .33 | .27 |
| Opportunistic/self-interest | 115.51** | 4 | .48 | .85 |
| Desperate | 254.57** | 4 | .71 | -71.40 |
| Delinquent | 80.36** | 4 | .40 | 0.47 |
| Wasteful | 256.27** | 4 | .72 | -48.60 |
| Unambitious | 57.10** | 4 | .34 | .29 |
| Classist | 253.11** | 4 | .71 | -120.13 |
| Extravagant | 239.93** | 4 | .69 | 21.67 |
| Impressionable | 49.87** | 4 | .32 | 0.25 |
| Compares themselves to lower groups | 68.53** | 4 | .37 | 0.38 |
| Unlucky | 316.50** | 4 | .80 | -4.83 |
| Distrustful | 4.20 | 4 | .09 | .02 |
| Frivolous | 214.54** | 4 | .66 | 5.88 |
| Hypocrite | 188.63** | 4 | .61 | 3.02 |
| Fear of the poor | 207.06** | 4 | .64 | 4.71 |
| Susceptible | 40.83** | 4 | .29 | .19 |

**Table S5**

*Bonferroni post hoc test of each negative trait showing the standardized residuals for testing independence for each group.*

| Negative Trait | Poor | Low and working classes | Middle classes | Upper classes | Rich and Beyond |  |
| --- | --- | --- | --- | --- | --- | --- |
| Haughty | 7.11** | 6.35** | 4.28** | -8.30** | -9.44** | No |
|  | -7.11** | -6.35** | -4.28** | 8.30** | 9.44** | Yes |
| Selfish | 6.86** | 6.95** | 4.58** | -7.95** | -10.43** | No |
|  | -6.86** | -6.95** | -4.58** | 7.95** | 10.43** | Yes |
| Class-unconscious | 3.77** | 4.73** | 0.05 | -2.50 | -6.04** | No |
|  | -3.77** | -4.73** | -0.05 | 2.50 | 6.04** | Yes |
| Conformist | 1.83 | -3.85** | -7.13** | 3.76** | 5.38** | No |
|  | -1.83 | 3.85** | 7.13** | -3.76** | -5.38** | Yes |
| Materialistic | 8.36** | 7.19** | 2.72 | -8.59** | -9.66** | No |
|  | -8.36** | -7.19** | -2.72 | 8.59** | 9.66** | Yes |
| Arrogant | 7.40** | 7.40** | 5.18** | -8.68** | -11.29** | No |
|  | -7.40** | -7.40** | -5.18** | 8.68** | 11.29** | Yes |
| Greedy | 6.86** | 6.48** | 3.97** | -7.25** | -10.06** | No |
|  | -6.86** | -6.48** | -3.97** | 7.25** | 10.06** | Yes |
| Economically concerned | -6.99** | -9.12** | -2.87* | 9.22** | 9.76** | No |
|  | 6.99** | 9.12** | 2.87* | -9.22** | -9.76** | Yes |
| Pretentious | 7.34** | 7.62** | 4.62** | -7.80** | -11.75** | No |
|  | -7.34** | -7.62** | -4.62** | 7.80** | 11.75** | Yes |
| Sad | -9.47** | -3.46** | 4.53** | 5.90** | 2.52 | No |
|  | 9.47** | 3.46** | -4.53** | -5.90** | -2.52 | Yes |
| Careless | -2.06 | 0.87 | 3.82** | -1.49 | -1.13 | No |
|  | 2.06 | -0.87 | -3.82** | 1.49 | 1.13 | Yes |
| Unsociable | -2.40 | 3.28* | 4.84** | 0.71 | -6.42** | No |
|  | 2.40 | -3.28* | -4.84** | -0.71 | 6.42** | Yes |
| Bad | 2.01 | 4.14** | 4.24** | -1.34 | -9.05** | No |
|  | -2.01 | -4.14** | -4.24** | 1.34 | 9.05** | Yes |
| Indifferent | 4.67** | 4.02** | -1.42 | -3.44** | -3.82** | No |
|  | -4.67** | -4.02** | 1.42 | 3.44** | 3.82** | Yes |
| Opportunistic/self-interest | 5.98** | 5.26** | 1.33 | -6.29** | -6.29** | No |
|  | -5.98** | -5.26** | -1.33 | 6.29** | 6.29** | Yes |
| Desperate | -12.74** | -5.97** | 4.76** | 7.17** | 6.79** | No |
|  | 12.74** | 5.97** | -4.76** | -7.17** | -6.79** | Yes |
| Delinquent | -6.38** | 2.31 | 5.92** | 2.04 | -3.89** | No |
|  | 6.38** | -2.31 | -5.92** | -2.04 | 3.89** | Yes |
| Wasteful | 7.53** | 6.68** | 5.07** | -7.61** | -11.67** | No |
|  | -7.53** | -6.68** | -5.07** | 7.61** | 11.67** | Yes |
| Unambitious | -3.10* | -4.49** | -1.33 | 4.31** | 4.61** | No |
|  | 3.10* | 4.49** | 1.33 | -4.31** | -4.61** | Yes |
| Classist | 8.09** | 8.00** | 2.80 | -8.99** | -9.91** | No |
|  | -8.09** | -8.00** | -2.80 | 8.99** | 9.91** | Yes |
| Extravagant | 5.98** | 7.02** | 5.60** | -6.97** | -11.62** | No |
|  | -5.98** | -7.02** | -5.60** | 6.97** | 11.62** | Yes |
| Impressionable | -1.99 | -4.25** | -1.99 | 2.98* | 5.24** | No |
|  | 1.99 | 4.25** | 1.99 | -2.98* | -5.24** | Yes |
| Compares themselves to lower groups | 6.76** | 0.44 | -5.69** | -2.52 | 1.00 | No |
|  | -6.76** | -0.44 | 5.69** | 2.52 | -1.00 | Yes |
| Unlucky | -13.93** | -7.17** | 5.93** | 7.72** | 7.44** | No |
|  | 13.93** | 7.17** | -5.93** | -7.72** | -7.44** | Yes |
| Distrustful | -1.21 | 0.50 | 1.65 | -0.04 | -0.91 | No |
|  | 1.21 | -0.50 | -1.65 | 0.04 | 0.91 | Yes |
| Frivolous | 5.88** | 7.01** | 4.48** | -6.10** | -11.28** | No |
|  | -5.88** | -7.01** | -4.48** | 6.10** | 11.28** | Yes |
| Hypocrite | 6.89** | 6.62** | 2.87* | -7.23** | -9.16** | No |
|  | -6.89** | -6.62** | -2.87* | 7.23** | 9.16** | Yes |
| Fear of the poor | 8.89** | 6.70** | 0.81 | -7.79** | -8.58** | No |
|  | -8.89** | -6.70** | -0.81 | 7.79** | 8.58** | Yes |
| Susceptible | -3.34** | -3.80** | 0.05 | 3.22** | 3.87** | No |
|  | 3.34** | 3.80** | -0.05 | -3.22** | -3.87** | Yes |

**Perception of social class in terms of positive valence traits.** The post hoc Bonferroni tests revealed the specific group and direction in which traits were associated (see Table S6). After inspecting the pattern of the association, we found that most of the positive traits were linked to “low and working classes” compared to the remaining groups. Contrary to expectations, the “poor” group did not have a higher percentage of positive traits (see Table S7).

**Table S6**

*Chi-square test showing the associations between groups (poor, low and working classes, middle classes, upper classes, and rich and beyond) and positive traits.*

| Positive Trait | χ2 | gl | Cramer’s V | odds ratio (OR) |
| --- | --- | --- | --- | --- |
| Humble | 235.01** | 4 | .69 | 14.70 |
| Kind | 140.34** | 4 | .53 | 1.27 |
| Happy | 92.31** | 4 | .43 | .58 |
| Value things | 285.15** | 4 | .76 | -8.35 |
| Solidary | 139.62** | 4 | .53 | 1.25 |
| Empathetic | 183.92** | 4 | .61 | 2.74 |
| Hardworking | 158.53** | 4 | .56 | 1.71 |
| High educated | 69.58** | 4 | .37 | .38 |
| Class-conscious | 46.58** | 4 | .31 | .23 |
| Distinct | 38.99** | 4 | .28 | .18 |
| Familiar | 125.49** | 4 | .50 | 1.00 |
| Competent | 74.83** | 4 | .39 | .42 |
| Approachable | 206.41** | 4 | .64 | 4.63 |
| Survivor | 283.71** | 4 | .75 | -8.67 |
| Sociable | 71.69** | 4 | .39 | .40 |
| Helpful | 170.97** | 4 | .58 | 2.14 |
| Competitive | 128.18** | 4 | .51 | 1.04 |
| Honest | 148.13** | 4 | .54 | 1.44 |
| Calm | 59.04** | 4 | .34 | .31 |
| Diverse | 75.40** | 4 | .39 | .43 |

**Table S7**

*Bonferroni post hoc test of each positive trait showing the standardized residuals for testing independence for each group.*

| Positive trait | Poor | Low and working classes | Middle classes | Upper classes | Rich and Beyond |  |
| --- | --- | --- | --- | --- | --- | --- |
| Humble | -5.91** | -9.23** | -2.97* | 8.47** | 9.64** | No |
|  | 5.91** | 9.23** | 2.97* | -8.47** | -9.64** | Yes |
| Kind | -.90 | -6.64** | -5.74** | 4.48** | 8.80** | No |
|  | .90 | 6.64** | 5.74** | -4.48** | -8.80** | Yes |
| Happy | 7.03** | 2.47 | -6.53** | -4.02** | 1.06 | No |
|  | -7.03** | -2.47 | 6.53** | 4.02** | -1.06 | Yes |
| Value things | -6.71** | -9.01** | -4.65** | 9.42** | 10.95** | No |
|  | 6.71** | 9.01** | 4.65** | -9.42** | -10.95** | Yes |
| Solidary | -.26 | -5.72** | -7.15** | 5.10** | 8.04** | No |
|  | .26 | 5.72** | 7.15** | -5.10** | -8.04** | Yes |
| Empathetic | -2.02 | -7.73** | -6.12** | 6.73** | 9.13** | No |
|  | 2.02 | 7.73** | 6.12** | -6.73** | -9.13** | Yes |
| Hardworking | .92 | -7.73** | -6.58** | 5.15** | 8.23** | No |
|  | -.92 | 7.73** | 6.58** | -5.15** | -8.23** | Yes |
| High educated | 4.85** | -1.15 | -6.69** | -1.05 | 4.03** | No |
|  | -4.85** | 1.15 | 6.69** | 1.05 | -4.03** | Yes |
| Class-conscious | -1.11 | -4.67** | -2.00 | 3.17** | 4.60** | No |
|  | 1.11 | 4.67** | 2.00 | -3.17** | -4.60** | Yes |
| Distinct | -1.80 | 2.16 | 3.34** | 1.53 | -5.23** | No |
|  | 1.80 | -2.16 | -3.34** | -1.53 | 5.23** | Yes |
| Familiar | -.94 | -5.68** | -6.12** | 4.80** | 7.95** | No |
|  | .94 | 5.68** | 6.12** | -4.80** | -7.95** | Yes |
| Competent | 5.33** | -1.36 | -7.01** | -.64 | 3.72** | No |
|  | -5.33** | 1.36 | 7.01** | .64 | -3.72** | Yes |
| Approachable | 0.50 | -8.19** | -8.03** | 6.70** | 9.02** | No |
|  | -0.50 | 8.19** | 8.03** | -6.70** | -9.02** | Yes |
| Survivor | -12.72** | -7.02** | 3.55** | 7.96** | 8.23** | No |
|  | 12.72** | 7.02** | -3.55** | -7.96** | -8.23** | Yes |
| Sociable | 3.60** | -2.60 | -6.74** | .18 | 5.29** | No |
|  | -3.60** | 2.60 | 6.74** | -.18 | -5.29** | Yes |
| Helpful | -2.52** | -8.47** | -4.24** | 6.76** | 8.48** | No |
|  | 2.52** | 8.47** | 4.24** | -6.76** | -8.48** | Yes |
| Competitive | 8.22** | 5.09** | -3.09* | -6.68** | -3.54** | No |
|  | -8.22** | -5.09** | 3.09* | 6.68** | 3.54** | Yes |
| Honest | -1.83 | -6.67** | -5.77** | 6.17** | 8.12** | No |
|  | 1.83 | 6.67** | 5.77** | -6.17** | -8.12** | Yes |
| Calm | 4.71** | 1.71 | -6.51** | -1.72 | 1.80 | No |
|  | -4.71** | -1.71 | 6.51** | 1.72 | -1.80 | Yes |
| Diverse | 4.71** | 1.71 | -6.51** | -1.71 | 1.80 | No |
|  | -4.71** | -1.71 | 6.51** | 1.71 | -1.80 | Yes |

**Study 3a**

**Feeling towards wealth-based groups: warm (or favorable) and cold (or unfavorable).** The repeated measures ANOVA revealed a main effect of the wealth-based group *F* (2,1456) = 1184 *p* <.001, η^2^ =.34.  Significant differences were found between rich group (*M* = 39.9, *SD* = 23.30) perceived as less warm (less favorable) compared to poor group (*M* = 65.60, *SD* = 21.10) or working class (*M* = 75.80, *SD* = 18.30), with *p* <.001 in both contrasts. Importantly, the working class was also perceived more warmly than the poor group.

**Study 3b**

The design of Study 3b closely mirrored that of Study 2. However, the word clouds used in Study 2 included terms that could convey implicit social class valences (e.g., "needy", "rotten with money") or terms that did not fit neatly into the proposed categories (e.g., "football player"). These terms could potentially inadvertently bolster class stereotypes. To address this issue, we developed more neutral stimuli for Study 3b.

Based on participants’ interaction with the wealth graph used during the interview in Study 1 (see Figure S8), we segmented the graph into 5 groups (Figure S9, S10, S11, S12, S13). The information shown to participant was “The graph shows how wealth is distributed, from those with the least wealth to those with the most, in ascending order", "The graph can be divided into different groups according to the wealth they have. For example, it can be segmented into 5 groups, ranging from Group 1 (the people with the least wealth) to Group 5 (the people with the most wealth), through Group 2, Group 3 and Group 4 in ascending order of wealth.". We deliberately omitted any specific terms referring to these groups or social classes. We then highlighted the boundaries between groups and asked participants to complete an affective thermometer.

**Figure S8.**

*Wealth distribution graph segmented by five wealth-based groups*


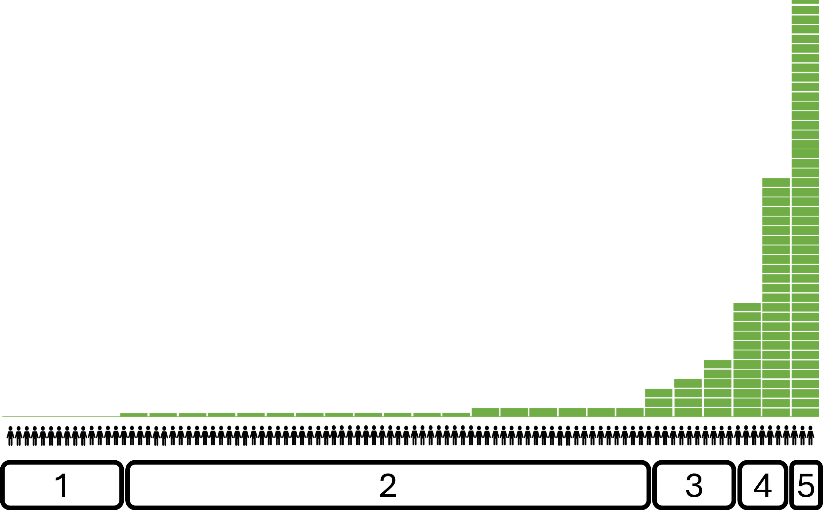


**Figure S9**

*Stimulus graph for Group 1*


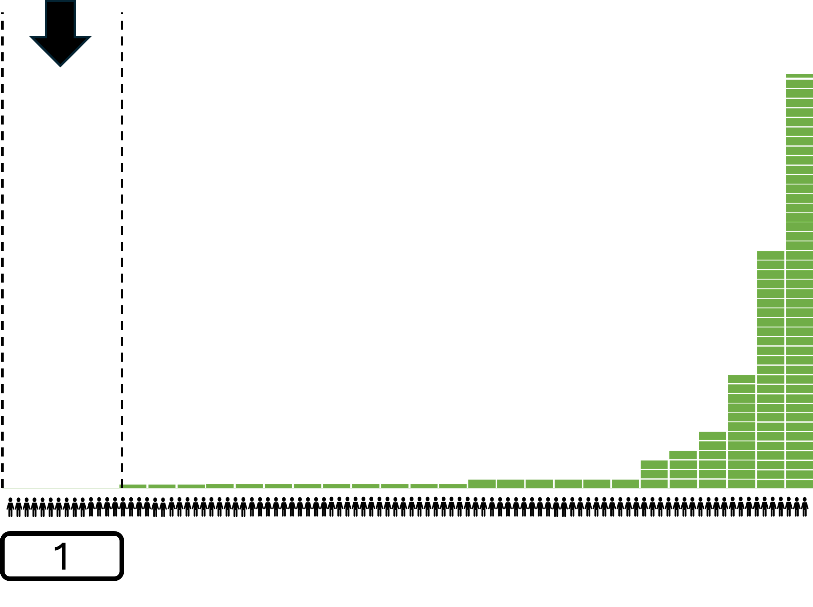


**Figure S10**.

*Stimulus graph for Group 2*


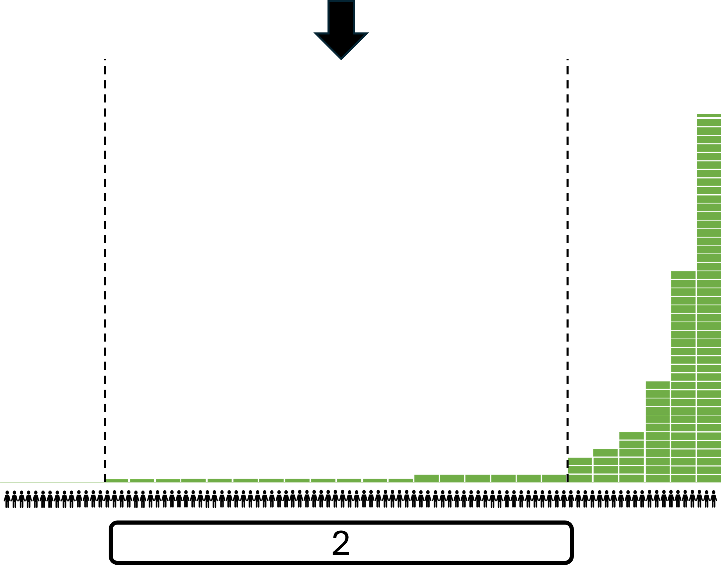


**Figure S11**.

*Stimulus graph for Group 3*


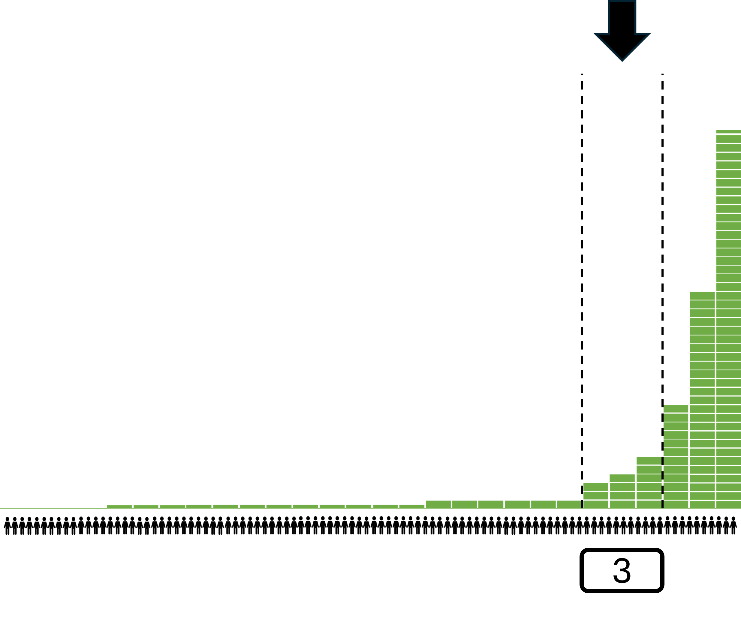


**Figure S12.**

*Stimulus graph for Group 4*


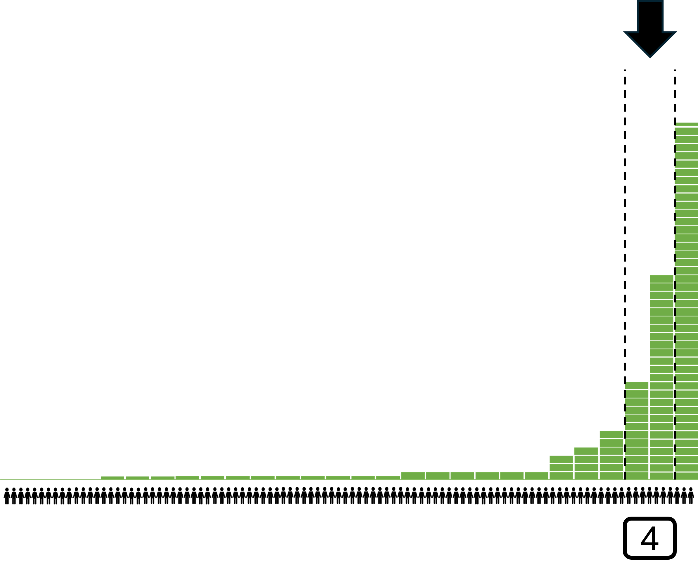


**Figure S13.**

*Stimulus graph for Group 5*


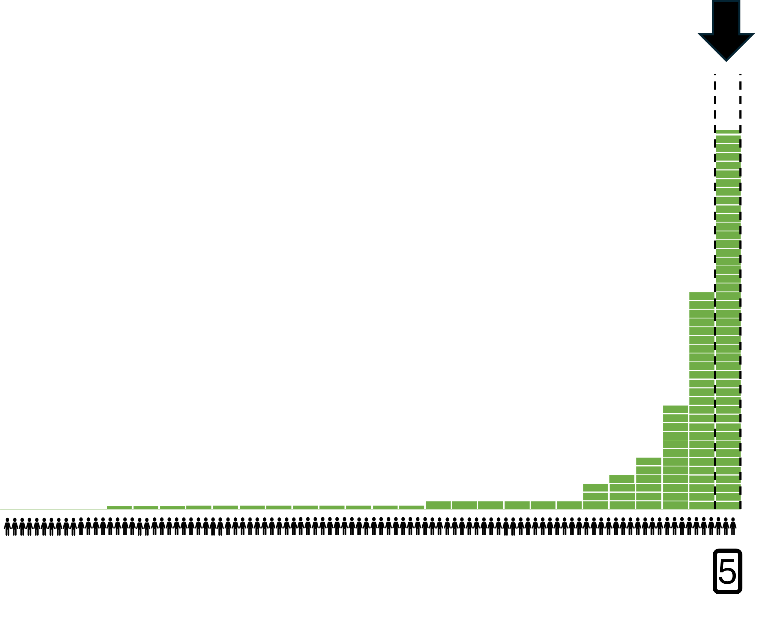


Additionally, we asked participants to name what they would call each wealth group. This step allowed us to verify that participants understood the procedure and the graph. We excluded 10 participants due to incorrect use of terms. Finally, participants evaluated the traits they associated with each group possessed and responded to the measures provided.

**Results**

**Feelings towards wealth-based groups: warm (favourable) and cold (unfavourable).** The repeated measures ANOVA revealed a main effect of wealth-based group *F* (4,189) = 93.1 *p* <.001, *η^2^* =.26. Significant differences were with Group 5 (*M* = 30, *SD* = 28.10) perceived as less warm compared to Group 1 (*M* = 60.50, *SD* = 24.40) and Group 2 (*M* = 67.30, *SD* = 21), both p <.001. Group 4 (*M* = 46.50, *SD* = 21.90) was also perceived as less warm than Group 1 and Group 2. However, Group 3 (*M* = 62.60; *SD* = 18.40) was perceived more favourably than Groups 4 and 5 (all *ps* <. 001), but similarly to Groups 1 and 2 (*p* = .999; *p* = 060). See Figure S13 in supplementary material.

**Figure S13.**

Distribution of affect scores across wealth groups


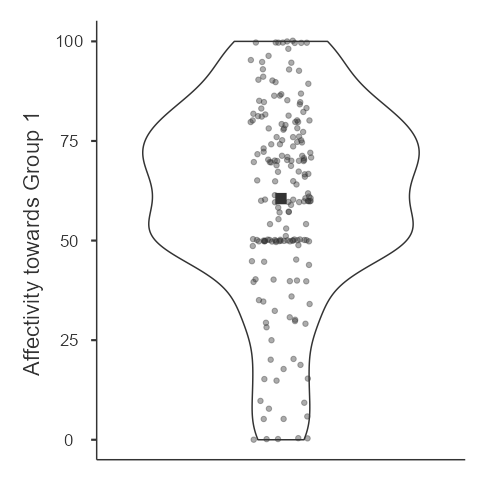

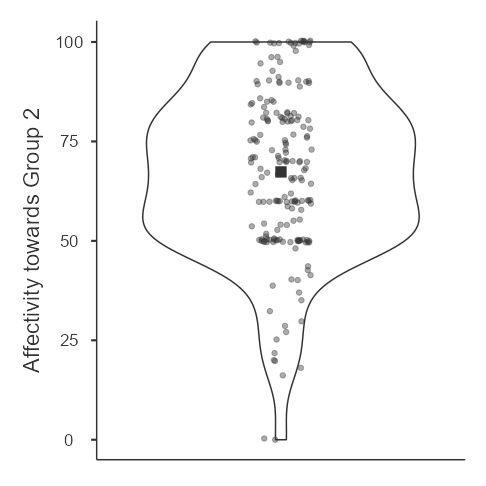


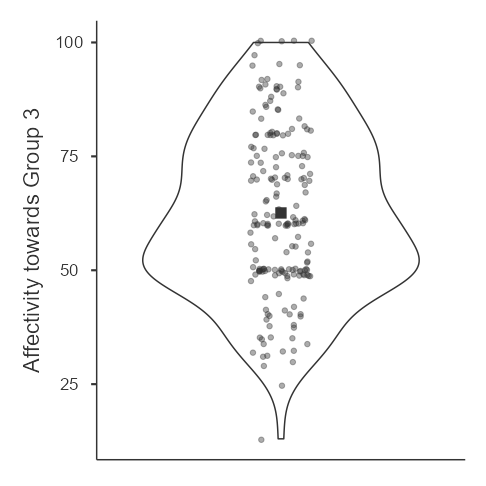

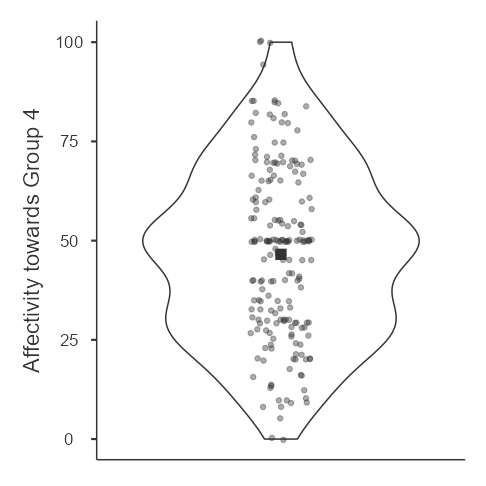


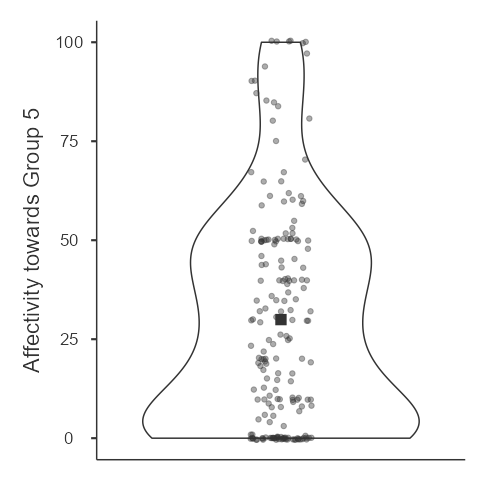


**Perception of wealth-based groups in terms of negative valence traits.** The post hoc Bonferroni tests revealed that all the associations between negative traits and wealth-based groups were statistically significant (Table S8). After inspecting the pattern of the association, we found that most of the negative traits were linked to rich-and-beyond and upper-class compared to the remaining group (Table S9).

**Table S8**

*Chi-square test showing the associations between groups (Group 1, Group 2, Group 3, Group 4 and Group 5) and negative traits*

| **Negative Trait** | **χ2** | **gl** | **Cramer’s V** |
| --- | --- | --- | --- |
| Haughty | 300.07** | 4 | .56 |
| Selfish | 388.65** | 4 | .64 |
| Class-unconscious | 32.73** | 4 | .19 |
| Conformist | 167.12** | 4 | .42 |
| Materialistic | 269.10** | 4 | .53 |
| Arrogant | 433.96** | 4 | .68 |
| Greedy | 355.76** | 4 | .61 |
| Economically concerned | 519.09** | 4 | .74 |
| Pretentious | 429.15** | 4 | .67 |
| Sad | 284.73** | 4 | .55 |
| Careless | 57.19** | 4 | .25 |
| Unsociable | 151.25** | 4 | .40 |
| Bad | 194.96** | 4 | .45 |
| Indifferent | 142.26** | 4 | .39 |
| Opportunistic/self-interest | 248.61** | 4 | .51 |
| Desperate | 559.44** | 4 | .77 |
| Delinquent | 134.44** | 4 | .38 |
| Wasteful | 202.61** | 4 | .46 |
| Unambitious | 177.51** | 4 | .43 |
| Classist | 403.88** | 4 | .65 |
| Extravagant | 402.58** | 4 | .65 |
| Impressionable | 177.73** | 4 | .43 |
| Compares themselves to lower groups | 135.79** | 4 | .38 |
| Unlucky | 571.01** | 4 | .77 |
| Distrustful | 49.95** | 4 | .23 |
| Frivolous | 414.42** | 4 | .66 |
| Hypocrite | 300.44** | 4 | .56 |
| Fear of the poor | 336.40** | 4 | .60 |
| Susceptible | 38.60** | 4 | .20 |

**Table S9**

*Bonferroni post hoc test of each negative trait showing the standardized residuals for testing independence for each group.*

| Negative Trait | Group 1 | Group 2 | Group 3 | Group 4 | Group 5 |  |
| --- | --- | --- | --- | --- | --- | --- |
| Haughty | 9.72** | 7.66** | 2.87* | -8.08** | -12.18** | No |
|  | -9.72** | -7.66** | -2.87* | 8.08** | 12.18** | Yes |
| Selfish | 8.81** | 8.14** | 6.14** | -7.21** | -15.88** | No |
|  | -8.81** | -8.14** | -6.14** | 7.21** | 15.88** | Yes |
| Class-unconscious | 2.04 | 2.38 | 1.87 | -1.19 | -5.11** | No |
|  | -2.04 | -2.38 | -1.87 | 1.19 | 5.11** | Yes |
| Conformist | 1.42 | -7.29** | -7.64** | 5.26** | 8.23** | No |
|  | -1.42 | **7.29**** | 7.64** | -5.26** | -8.23** | Yes |
| Materialistic | 10.06** | 6.81** | 2.27 | -8.93** | -10.23** | No |
|  | -10.06** | -6.81** | 2.27 | 8.93** | 10.23** | Yes |
| Arrogant | 9.30** | 9.64** | 5.35** | -7.68** | -16.60** | No |
|  | -9.30** | -9.64** | -5.35** | 7.68** | 16.60** | Yes |
| Greedy | 8.64** | 7.78** | 6.24** | -8.67** | -13.98** | No |
|  | -8.64** | -7.78** | -6.24** | 8.67** | 13.98** | Yes |
| Economically concerned | -11.33** | -12.96** | -2.07 | 12.70** | 13.67** | No |
|  | 11.33** | **12.96**** | 2.07 | -12.70** | -13.67** | Yes |
| Pretentious | 9.43** | 8.92** | 6.01** | -7.99** | -16.37** | No |
|  | -9.43** | -8.92** | -6.01** | 7.99** | 16.37** | Yes |
| Sad | -13.62** | -6.05** | 5.30** | 8.26** | 6.12** | No |
|  | 13.62** | **6.05**** | -5.30** | -8.26** | -6.12** | Yes |
| Careless | -6.61** | 0.63 | 4.81** | 2.00 | 0.42 | No |
|  | 6.61** | -0.63 | -4.81** | -2.00 | -0.42 | Yes |
| Unsociable | -1.62 | 5.19** | 6.48** | 0.77 | -10.82** | No |
|  | 1.62 | -5.19** | -6.48** | -0.77 | 10.82** | Yes |
| Bad | 1.40 | 5.36** | 6.27** | 0.14 | -13.18** | No |
|  | -1.40 | -5.36** | -6.27** | -0.14 | 13.18** | Yes |
| Indifferent | 6.29** | 6.80** | -0.58 | -3.68** | -8.83** | No |
|  | -6.29** | -6.80** | 0.58 | 3.68** | 8.83** | Yes |
| Opportunistic/self-interest | 7.37** | 7.70** | 3.93 | -8.68** | -10.31** | No |
|  | -7.37** | -7.70** | -3.93 | 8.68** | 10.31** | Yes |
| Desperate | -19.39** | -8.07** | 7.04** | 9.95** | 10.47** | No |
|  | 19.39** | **8.07**** | -7.04** | -9.95** | -10.47** | Yes |
| Delinquent | -8.37** | 2.05 | 7.59** | 3.56** | -4.84** | No |
|  | 8.37** | -2.05 | -7.59** | -3.56** | 4.84** | Yes |
| Wasteful | 7.72** | 5.33** | 3.28* | -4.92** | -11.41** | No |
|  | -7.72** | -5.33** | -3.28* | 4.92** | 11.41** | Yes |
| Unambitious | -4.27* | -8.94** | -2.15 | 7.35** | 8.05** | No |
|  | 4.27* | **8.94**** | 2.15 | -7.35** | -8.05** | Yes |
| Classist | 10.15** | 9.32** | 4.34 | -9.42** | -14.40** | No |
|  | -10.15** | -9.32** | -4.34 | 9.42** | 14.40** | Yes |
| Extravagant | 6.91** | 8.69** | 6.55** | -4.31** | -17.84** | No |
|  | -6.91** | -8.69** | -6.55** | 4.31** | 17.84** | Yes |
| Impressionable | -4.51** | -7.92** | -3.21** | 6.04** | 9.61** | No |
|  | 4.51** | **7.92**** | 3.21** | -6.04** | -9.61** | Yes |
| Compares themselves to lower groups | 7.47** | -0.98 | -9.44** | -1.66 | 4.59** | No |
|  | -7.47** | -0.98 | 9.44** | 1.66 | -4.59** | Yes |
| Unlucky | -19.62** | -8.23** | 7.92** | 9.96** | 9.96** | No |
|  | 19.62** | **8.23**** | -7.92** | -9.96** | -9.96** | Yes |
| Distrustful | -1.56 | 3.32** | 4.46** | -0.91 | -5.31** | No |
|  | 1.56 | -3.32** | -4.46** | 0.91 | 5.31** | Yes |
| Frivolous | 9.01** | 8.83** | 6.10** | -7.77** | -16.16** | No |
|  | -9.01** | -8.83** | -6.10** | 7.77** | 16.16** | Yes |
| Hypocrite | 8.73** | 7.73** | 4.06** | -7.76** | -12.76** | No |
|  | -8.73** | -7.73** | -4.06** | 7.76** | 12.76** | Yes |
| Fear of the poor | 10.76** | 8.61** | 1.18 | -9.44** | -11.76** | No |
|  | -10.76** | -8.61** | -1.18 | 9.44** | 11.76** | Yes |
| Susceptible | -3.78** | -3.45** | 0.66 | 3.12** | 3.44** | No |
|  | 3.78** | **3.45**** | -0.66 | -3.12** | -3.44** | Yes |

**Perception of social class in terms of positive valence traits.** The post hoc Bonferroni tests revealed the specific group and direction in which traits were associated (see Table S10). After inspecting the pattern of the association, we found that most of the positive traits were linked to “low and working classes” compared to the remaining groups. Contrary to expectations, the “poor” group did not have a higher percentage of positive traits (see Table S11).

**Table S10**

*Chi-square test showing the associations between groups (Group 1, Group 2, Group 3, Group 4 and Group 5) and positive traits.*

| Positive Trait | χ2 | gl | Cramer’s V |
| --- | --- | --- | --- |
| Humble | 468.28** | 4 | .72 |
| Kind | 244.30** | 4 | .51 |
| Happy | 165.72** | 4 | .42 |
| Value things | 309.67** | 4 | .57 |
| Solidary | 187.33** | 4 | .44 |
| Empathetic | 281.83** | 4 | .54 |
| Hardworking | 280.23** | 4 | .54 |
| High educated | 111.18** | 4 | .34 |
| Class-conscious | 29.33** | 4 | .18 |
| Distinct | 128.94** | 4 | .37 |
| Familiar | 174.53** | 4 | .43 |
| Competent | 147.03** | 4 | .39 |
| Approachable | 313.66** | 4 | .57 |
| Survivor | 493.06** | 4 | .72 |
| Sociable | 174.16** | 4 | .43 |
| Helpful | 298.74** | 4 | .56 |
| Competitive | 216.69** | 4 | .48 |
| Honest | 209.06** | 4 | .47 |
| Calm | 92.77** | 4 | .31 |
| Diverse | 173.19** | 4 | .43 |

**Table S11**

*Bonferroni post hoc test of each positive trait showing the standardized residuals for testing independence for each group.*

| Positive trait | Group 1 | Group 2 | Group 3 | Group 4 | Group 5 |  |
| --- | --- | --- | --- | --- | --- | --- |
| Humble | -11.46** | -12.59** | -1.07 | 11.75** | 13.37** | No |
|  | 11.46** | 12.59** | 1.07 | -11.75** | -13.37** | Yes |
| Kind | -2.09 | -7.98** | -7.98** | 6.74** | 11.32** | No |
|  | 2.09 | 7.98** | **7.98**** | -6.74** | -11.32** | Yes |
| Happy | 10.09** | 4.32** | -7.16** | -5.86** | -1.30 | No |
|  | -10.09** | -4.32** | **7.16**** | 5.86** | 1.30 | Yes |
| Value things | -6.46** | -9.73** | -4.83** | 8.71** | 12.31** | No |
|  | 6.46** | 9.73** | **4.83**** | -8.71** | -12.31** | Yes |
| Solidary | -1.11 | -7.77** | -6.80** | 6.54** | 9.14** | No |
|  | 1.11 | 7.77** | **6.80**** | -6.54** | -9.14** | Yes |
| Empathetic | -3.50** | -9.51** | -6.91** | 8.66** | 11.26** | No |
|  | 3.50** | 9.51** | **6.91**** | -8.66** | -11.26** | Yes |
| Hardworking | 1.29 | -9.04** | -8.71** | 7.29** | 11.74** | No |
|  | 1.29 | 9.04** | **8.71**** | -7.29** | -11.74** | Yes |
| High educated | 8.04** | -1.30 | -7.31** | -2.80 | 3.37** | No |
|  | -8.04** | 1.30 | **7.31**** | 2.80 | -3.37** | Yes |
| Class-conscious | -.75 | -3.84** | -1.40 | 2.18 | 3.82** | No |
|  | .75 | 3.84** | 1.40 | -2.18 | -3.82** | Yes |
| Distinct | -2.54 | 3.73** | **6.70**** | 1.75 | -9.64** | No |
|  | 2.54 | -3.73** | -6.70** | -1.75 | 9.64** | Yes |
| Familiar | -2.06 | -7.86** | -5.21** | 5.24** | 9.88** | No |
|  | 2.06 | 7.86** | **5.21**** | -5.24** | -9.88** | Yes |
| Competent | 8.03** | -2.17 | -8.75** | -2.67 | 5.56** | No |
|  | -8.03** | 2.17 | **8.75**** | 2.67 | -5.56** | Yes |
| Approachable | -1.98 | -9.93** | -8.47** | 7.91** | 12.45** | No |
|  | 1.98 | 9.93** | **8.47**** | -7.91** | -12.45** | Yes |
| Survivor | -17.71** | -7.76** | 4.35** | 10.32** | 10.81** | No |
|  | 17.71** | 7.76** | -4.35** | -10.32** | -10.81** | Yes |
| Sociable | .37 | -7.20** | -7.20** | 4.24** | 9.79** | No |
|  | -.37 | 7.20** | **7.20**** | -4.24** | -9.79** | Yes |
| Helpful | -5.98** | -10.59** | -3.85** | 9.14** | 11.27** | No |
|  | 5.98** | 10.59** | **3.85**** | -9.14** | -11.27** | Yes |
| Competitive | 10.70** | 6.80** | -3.77** | -7.84** | -5.88** | No |
|  | -10.70** | -6.80** | **3.77**** | 7.84** | 5.88** | Yes |
| Honest | -3.57** | -8.44** | -5.03** | 6.65** | 10.38** | No |
|  | 3.57** | 8.44** | **5.03**** | -6.65** | -10.38** | Yes |
| Calm | 7.14** | 2.60 | -6.98** | -3.08 | .32 | No |
|  | -7.14** | -2.60 | **6.98**** | 3.08 | -.32 | Yes |
| Diverse | -2.21 | -7.24** | -5.94** | 6.07** | 9.32** | No |
|  | 2.21 | 7.24** | **5.94**** | -6.07** | -9.32** | Yes |
